# Sharing Pollinators and Viruses: Virus Diversity of Pollen in a Co-Flowering Community

**DOI:** 10.1093/icb/icaf073

**Published:** 2025-06-02

**Authors:** Andrea M Fetters, Paul G Cantalupo, Maria Teresa Sáenz Robles, James M Pipas, Tia-Lynn Ashman

**Affiliations:** Department of Biological Sciences, University of Pittsburgh, 4249 Fifth Avenue, Pittsburgh, PA 15260, USA; Department of Evolution, Ecology, and Organismal Biology, The Ohio State University, 318 W. 12th Avenue, Columbus, OH 43210, USA; Department of Biological Sciences, University of Pittsburgh, 4249 Fifth Avenue, Pittsburgh, PA 15260, USA; Department of Biomedical Informatics, University of Pittsburgh, 5607 Baum Boulevard, Pittsburgh, PA 15206, USA; Department of Biological Sciences, University of Pittsburgh, 4249 Fifth Avenue, Pittsburgh, PA 15260, USA; Department of Biological Sciences, University of Pittsburgh, 4249 Fifth Avenue, Pittsburgh, PA 15260, USA; Department of Biological Sciences, University of Pittsburgh, 4249 Fifth Avenue, Pittsburgh, PA 15260, USA

## Abstract

Co-flowering plant species frequently share pollinators, flower-inhabiting bacteria, and fungi, but whether pollen-associated viruses are shared is unknown. Given that pollen-associated viruses are sexually transmitted diseases, their diversity is expected to increase with pollinator sharing. We conducted a metagenomic study to identify pollen-associated viruses from 18 co-flowering plant species to determine whether (1) life history, floral traits, or pollination generalism were associated with viral richness, and (2) plants shared pollen-associated viruses. We demonstrated that pollination generalism influences pollen-associated virus richness and the extent of pollen virus sharing between plant species. We also revealed that perenniality, multiple flowers, and bilateral floral symmetry were associated with high pollen viral richness locally, confirming and extending patterns observed previously at a continental scale. Our results highlight the importance of plant-pollinator interactions as drivers of plant-viral interaction diversity.

## Introduction

In plant communities, co-flowering species often share pollinators (e.g., Johnson and Steiner 2000; Bascompte et al. 2006). Pollinator sharing can have fitness benefits, such as facilitation of pollinator visits (e.g., Moeller 2004), or have costs, such as the loss of reproductive opportunities via pollen misplacement (e.g., Moreira-Hernández and Muchhala 2019; Wei et al. 2021a). Recent studies have revealed that sharing pollinators leads to transfer of flower-, pollen-, and pollinator-associated microbes between plants (of the same or different species), as well as between pollinators themselves (Vannette 2020; Prosemans et al.^2021^; Steffan et al. 2024). Given the ubiquity, diversity, and impacts of the microbes associated with pollinators and flowering plants (Rebolleda-Gómez et al. 2019; Vannette 2020; and reviewed in Cullen et al. 2021a), it has been suggested that this tripartite interaction underlies phenotypic diversity and structure of terrestrial communities (Fetters and Ashman 2023; Steffan et al. 2024). While much of this work has focused on bacteria and fungi that inhabit nectar or petal surfaces (reviewed in Vannette 2020; Cullen et al. 2021a), viruses, especially pollen-associated ones, also are transferred by pollinators from flower to flower and use flowers as entry points for transmission (reviewed in Fetters and Ashman 2023). Thus, knowledge of the distribution, diversity, and impact of pollen-associated viruses is important to understanding how they too contribute to tripartite plant-pollinator-microbe interactions (Fetters and Ashman 2023).

Pollen‐associated viruses can reside on the outside or the inside of pollen grains (e.g., Hamilton et al. 1977; Isogai et al. 2014). They can infect plants through vertical (i.e., father [pollen] to offspring) and/or horizontal (i.e., individual to individual) pathways (Card et al. 2007). For instance, pollen transferred from an infected plant to the stigmas of an uninfected plant can result in infection of the maternal plant and/or its seeds, and this can occur within species and between species. Because transport is mediated by pollinators, viral spread is expected to be determined by the same floral traits that dictate pollinator attraction and “fit” and pollen collectability and transportability (Proesmans et al. 2021; Fetters and Ashman 2023). However, most of the research on pollen-associated viruses has examined cultivated plants in agricultural settings (often focusing on mechanisms of pathogenesis, e.g., Isogai et al. 2014), or bee-collected pollen (e.g., Tiritelli et al. 2024), leaving our understanding of the diversity and distribution of viruses in wild plants still in its infancy (reviewed in Fetters and Ashman 2023).

An exception is the recent study with 24 wild plant species across the United States of America: Fetters et al. (2022) found that pollen-associated viral taxa have broad host ranges, infecting wild plant taxa across a range of locations. In addition, in a phylogenetically controlled analysis, they revealed species with a high diversity of pollen-associated viruses had multiple-flowered inflorescences, bilaterally symmetric flowers, and less accessible floral rewards, as well as smaller, spiky pollen grains. Fetters et al. (2022) concluded that the association of viral diversity with these particular floral traits highlighted the need to incorporate plant-pollinator interactions as a driver of plant viral diversity.

Since pollen-associated viruses are transmitted during mating, they are sexually transmitted diseases. Thus, one might predict a similar positive association between a high probability of contact (e.g., promiscuity, many reproductive events) and high probability of obtaining the disease agent (Hamilton 1990; Wennström et al. 2003; Fetters and Ashman 2023). Specifically, plant species visited by numerous pollinator taxa (pollination generalist plants) or visited by pollinators that visit many plants species (generalist pollinators) or have many reproductive episodes (perennial life history), might encounter more pollen‐associated viruses than plant species that are visited by few pollinators (pollination specialists), have few reproductive episodes (annual life history), or those that do not rely on pollinators (are obligately autonomously self-pollinating). A similar role for pollinator generalism (greater diet breadth) has been proffered to explain pathogen exposure and/or microbial partner diversity, but this has received mixed support, perhaps due to compensating behavioral adaptations (Rothman et al. 2020; Keller et al. 2021; Nicholls et al. 2022). Nevertheless, the association between plant-pollination generalism/specialism and the virus diversity of pollen has not yet been directly tested (Fetters and Ashman 2023). Moreover, the extent of variation in pollen virus richness among plants species within a single co-flowering natural community remains unexplored. Thus, we do not know whether co-flowering community members that share pollinators also share pollen-associated viral taxa.

To address these gaps, we evaluated the richness of pollen-associated viruses of 18 co-flowering plant species from the serpentine seep community of McLaughlin Natural Reserve, Lower Lake, CA, USA. This community is characterized by high plant species diversity and endemism (Safford et al. 2005) and hosts a broad diversity of pollinators (Wei et al. 2021a). We performed metagenomic analyses of pollen to answer the following questions: (1) does taxonomic richness of viruses associated with pollen correlate with species-level floral, pollen, or life history traits? We were especially interested in determining whether traits previously revealed as important across species at the continental scale (e.g., inflorescence size, floral symmetry, and reward accessibility [Fetters et al. 2022]) were also salient at the local scale within a single community; (2) does taxonomic richness of pollen-associated viruses increase with pollination generalism, measured directly as the diversity of pollinators visiting the plant species?; and (3) do co-flowering plant species share pollen-associated viruses, and does the extent of sharing correlate with pollen virus or pollinator diversity?

## Methods

### Study system

Eighteen plant species (Table 1) of the serpentine seep meta-community of McLaughlin Natural Reserve, Lower Lake, CA, USA (38.8582°N, 122.4093°W) were the subject of this study. The aggregate of serpentine seeps at this location has been described as a metacommunity because their close geographic proximity (0.3–4 km linear distance between any two seeps; Koski et al. 2015) is within the range of foraging distance for many solitary and social bees (Beekman and Ratnieks 2000; Zurbuchen et al. 2010), which determines local plant colonization/extinction dynamics (Harrison et al. 2000). The seeps have unique soil chemistry and hydrology relative to the surrounding non-serpentine grassland (Koski et al. 2015; Arceo-Gómez et al. 2018; LeCroy et al. 2021; Wei et al. 2021a; Cullen et al. 2021b) that leads to significant co-flowering within them (Arceo-Gómez et al. 2016). The 18 species are morphologically diverse, herbaceous annuals or perennials, and are visited by a wide array of insect taxa (Koski et al. 2015; Wei et al. 2021a). The plant species studied herein are from the same set of serpentine seeps from which plant and pollinator data were collected by Wei et al. (2021a).

**Table 1:** Plant traits (and PC1, PC2), pollinator diversity, estimates of pollen-associated virus richness, virus sharing, and plant species with which viruses are shared.

	Pollinator attraction		Pollinator fit				Pollen grain collectability								
Plant species, 4-letter species code	Single flower?^1^	Flower size (mm)^2^	Flower restricted?^3^	Flower shape^4^	Flower symmetry	Flower tube length (mm)^5^	Pollen grain texture^6^	Pollen grain length (µm)^7^	Pollinator diversity^8^	Life history	PC1^9^	PC2^10^	Conservative estimate^11^	Relaxed estimate^12^	No. shared viruses: plant species^13^
*Agoseris heterophylla*, AGHE	No	10.07	No	Open	Bilateral	2.94	Spiky	31.74	2.34	Annual	−1.86	1.99	2	14	2: *E. lanatum* 2: *L. californica* 1: *M. guttatus*
*Anagallis arvensis*, ANAR	Yes	8.32	No	Open	Radial	0	Smooth	26.80	1.84	Annual	−1.54	−0.95	0	0	0
*Calochortus luteus*, CALU	Yes	39.37	No	Open	Radial	0	Smooth	57.89	3.00	Perennial	−0.88	−2.16	1	6	1: *C. gracilis* 1: *D. uliginosum* 3: *E. lanatum* 1: *L. californica* 1: *S. diploscypha*
*Castilleja rubicundula*, CARU	No	6.93	Yes	Closed	Bilateral	14.17	Smooth	30.79	2.61	Perennial	1.83	2.00	3	3	0
*Clarkia concinna*, CLCO	No	37.08	Yes	Closed	Bilateral	17.33	Smooth	104.73	2.66	Annual	2.92	0.59	0	1	1: *E. lanatum* 1: *L. californica* 1: *L. dichotomus*
*Clarkia gracilis*, CLGR	Yes	40.10	Yes	Open	Radial	9.54	Smooth	154.60	2.62	Annual	1.38	−2.53	1	4	1: *C. luteus* 1: *D. uliginosum* 1: *E. lanatum*
*Delphinium uliginosum*, DEUL	No	30.18	Yes	Closed	Radial	12.11	Smooth	30.81	2.24	Perennial	1.89	0.08	3	9	1: *C. gracilis* 1: *C. luteus* 1: *E. lanatum*
*Eriophyllum lanatum*, ERLA	No	4.80	No	Open	Bilateral	2.39	Spiky	34.21	3.99	Perennial	−1.99	2.12	3	11	2: *A. heterophylla* 1: *C. concinna* 1: *C. gracilis* 3: *C. luteus* 1: *D. uliginosum* 2: *L. californica* 1: *L. dichotomus* 1: *S. diploscypha*
*Eschscholzia californica*, ESCA	Yes	32.43	No	Open	Radial	4.32	Smooth	19.26	2.86	Annual	−0.79	−1.56	1	4	1: *L. californica* 1: *S. bellum*
*Lasthenia califórnica*, LACA	No	2.71	No	Open	Bilateral	1.47	Spiky	14.80	3.30	Perennial	−2.18	2.31	3	14	2: *A. heterophylla* 1: *C. concinna* 1: *C. luteus* 1: *E. califórnica* 2: *E. lanatumm* 2: *L. dichotomus* 1: *S. bellum*
*Leptosiphon bicolor*, LEBI (*Linanthus bicolor*)	Yes	9.25	Yes	Closed	Radial	15.15	Smooth	40.40	1.95	Annual	1.85	−0.22	1	4	0
*Linanthus dichotomus*, LIDI	Yes	26.71	Yes	Closed	Radial	9.26	Smooth	34.16	2.37	Annual	1.63	−0.86	0	3	1: *C. concinna* 1: *E. lanatum* 2: *L. californica*
*Mimulus guttatus*, MIGU (*Erythranthe guttata*)	No	24.44	Yes	Closed	Bilateral	13.60	Smooth	34.87	2.50	Annual	2.10	1.40	3	15	1: *A. heterophylla* 5: *M. nudatus*
*Mimulus nudatus*, MINU (*Erythranthe nudata*)	Yes	13.84	Yes	Closed	Bilateral	9.24	Smooth	33.14	2.85	Annual	1.56	0.67	1	8	5: *M. guttatus*
*Ranunculus californicus*, RACA	Yes	11.04	No	Open	Radial	0	Smooth	28.71	2.81	Perennial	−1.49	-1.05	0	11	1: *Z. venenosus*
*Sidalcea diploscypha*, SIDI	No	29.81	No	Open	Radial	0	Spiky	105.70	2.62	Annual	−1.64	-0.37	0	1	1: *C. luteus* 1: *E. lanatum*
*Sisyrinchium bellum*, SIBE	Yes	17.79	No	Open	Radial	0	Smooth	42.64	3.02	Perennial	−1.31	−1.36	2	6	1: *E. californica* 1: *L. californica*
*Zigadenus venenosus*, ZIVE (*Toxicoscordion venenosum)*	No	10.53	No	Open	Radial	0	Smooth	34.09	1.64	Perennial	−1.50	−0.10	0	1	1: *R. californicus*

^1^Single flower?: type of floral display presented by a plant species; no = multiple-flowered, yes = single-flowered, or flowers widely spaced on a stem.

^2^Flower size (mm): diameter of the flowers of a plant species, measured across their longest length.

^3^Flower restricted?: whether the flower shape of a plant species restricts access to its floral rewards.

^4^Flower shape: general morphology of the flowers of a plant species.

^5^Flower tube length: distance from the ovaries to the beginning of the flower tube (petal separation) of the flowers of a plant species; 0 for plant species whose flowers do

not have tubes.

^6^Pollen grain texture: texture of the pollen grain exines of a plant species; spiky = echinate, smooth = granulate (rough, but not spiky) or psilate.

^7^Pollen grain length (mm): diameter of the pollen grains of a plant species, measured across their longest length.

^8^Pollinator Shannon diversity: derived from multiple observations per week from April to June of 2016 and 2017.

^9,10^PC1, PC2: floral and pollen grain trait PC values for each plant species.

^11,12^Conservative, relaxed estimates: estimates of pollen virus richness of each plant species.

^13^Number shared viruses: plant species: the number of viruses each plant species is estimated to be sharing with other co-flowering plant species in the community; known viruses were determined to be shared when they were found in more than one plant species; novel viral genomes and variants were considered to be shared when their RdRps were 80–100% identical, or if their % identities were above the International Committee for the Taxonomy of Viruses (ICTV, 2025) family-specific species demarcation threshold.

### Plant traits and pollination generalism

Eight floral traits (Table 1) measured on ten flowers per species by Wei et al. (2021a) were used herein. We sought to determine whether those considered important for pollinator attraction, fit, and pollen grain collectability predicted pollen-associated virus richness (see Supplementary Table S1 for details). For our analysis, we coded the levels of each categorical trait as 0 or 1 as follows: inflorescence type (single flower or flowers spaced far apart on a stem vs. multiple flowers), flower restrictiveness (unrestrictive vs. restrictive), flower shape (open [aster-like] vs. closed [labiate or salverform]), flower symmetry (radial vs. bilateral), and pollen grain texture (smooth [psilate or granulate] vs. spiky [echinate]). A value of 1 reflects the state predicted to lead to higher pollen-associated virus richness. Continuous traits were flower size, flower tube length, and pollen grain length. All traits were standardized (i.e., mean = 0, standard deviation = 1), and we performed a principal component analysis (PCA) using the “prcomp” function in base R. It yielded two dominant principal components (PC1–2; Table 1; Supplementary Table S2), which together explained 69% of the phenotypic variation. We also considered the plant life history (i.e., annual vs. perennial), coded as 0 (annual [one reproductive episode]) or 1 (perennial [multiple reproductive episodes]).

The Shannon diversity of pollinator taxa (i.e., pollinator diversity) visiting each plant species (Table 1) was obtained from the study of Wei et al. (2021a). These data were derived from multiple observations per week between April and June of 2016 and 2017 at the multiple seeps within this meta-community. Insects were identified to species, or the lowest possible taxonomic level. High diversity reflects high pollination generalism (Dormann 2011).

### Pollen collection for virus detection

Following previous work (Fetters et al. 2022), we used sterile sonication techniques to collect 30–50 mg pollen (20 Hz; Fisherbrand Model 50, Fisher Scientific, Waltham, MA, USA) from newly dehiscing anthers from freshly opened flowers in the field (one sample per plant species, created by pooling pollen from 5 to 145 flowers from 4 to 110 plants per species; Supplementary Table S3). Pooling was necessary to achieve the required 30–50 mg of pollen for successful RNA extraction and sequencing, and given that different species produce different amounts of pollen per flower, the number of flowers pooled varied among species. The sampled plants were in full bloom, visually asymptomatic, abundant, and flowering at the time of pollen collection (May 2018). The pollen was stored in 2-mL Lysing Matrix D tubes (MP Biomedicals, Irvine, CA, USA) and kept frozen at −80°C until processed. For additional collection and extraction protocol details, see Fetters (2021).

### Pollen RNA extraction and sequencing

We focused on RNA viruses because most plant viruses have RNA genomes (Hulo et al. 2011; ICTV 2025). To detect all the pollen-associated viruses, we freeze-dried and disrupted the grains as in Fetters et al. (2022). We extracted the total RNA using the Quick-RNA Plant Miniprep Extraction Kit (Zymo Research Corporation, Irvine, CA, USA) following the manufacturer’s protocol.

We assessed the quality of the RNA using a NanoDrop Spectrophotometer 2000 (ThermoFisher Scientific, Waltham, MD, USA), the concentration with a Qubit 3.0 fluorometer (Invitrogen, ThermoFisher Scientific, Waltham, MA, USA), and the quality for high-throughput sequencing through TapeStation analysis at the Genomics Research Core (GRC) at the University of Pittsburgh. Only samples with RNA integrity values of at least 1.7 were sequenced (Supplementary Table S3). A stranded RNA library was created for each sample using the TruSeq Total RNA Library Kit (Illumina, Inc., San Diego, CA, USA) and ribosomally depleted using the RiboZero Plant Leaf Kit (Illumina, Inc., San Diego, CA, USA) at the GRC. Libraries from six plant species per sequencing run were pooled on one lane of an Illumina NextSeq500 platform. We sequenced to a depth of 127–205 million 75 bp paired-end reads per sample (Supplementary Table S3), and raw sequences were demultiplexed and trimmed of adapters.

### Pollen-associated virus detection and identification

Following Fetters et al. (2022), we used Pickaxe, a viral discovery pipeline, and several criteria to detect and identify known and novel pollen-associated viruses in each plant species. Briefly, Pickaxe aligned sequences (using the Bowtie2 aligner with default parameters, v2.3.4.2–3; Langmead and Salzberg 2012) to customized subtraction libraries (Supplementary Table S4), leaving only non-plant (i.e., viral) reads. Reads were assembled into contigs using the CLC Assembly Cell (Qiagen Digital Insights, Redwood City, CA, USA), and contigs were extended when there was overlap between them. Known viruses were identified via alignments between non-plant reads (21–6111 per plant species; Supplementary Table S3) and Viral RefSeq (VRS; Index of/refseq/release/viral (nih.gov)). Novel viral genomes were discovered by aligning the contigs (1–58 per plant species; Supplementary Table S3) to the NCBI nucleotide and protein databases with BLAST or RAPSearch2 (Zhao et al. 2012) algorithms.

Following the criteria set forth in our past work (Fetters et al. 2022), we considered a known virus to be present in the pollen only if the viral reads covered at least 20% of the top hit of an alignment with VRS and aligned to the top hit at least ten times. We also delineated novel viral genomes at the family level based upon the contig or extended contig length, % similarity to the top BLAST or RAPSearch2 hit, open reading frame (ORF) architecture as determined by ORFfinder searches with default parameters (NCBI; Home—ORFfinder—NCBI (nih.gov)), and conserved domain (i.e., proteins; CDs) presence verified by searching the Conserved Domain Database with default parameters (NCBI Conserved Domain Search (nih.gov)). All “coding-complete” novel viral genomes or variants are those whose lengths, ORF architecture, and CDs matched their putative viral family. Any that did not meet these criteria were called “partial.” We noted species-specific RNA-dependent RNA polymerase (RdRp) from *all* sequences, as they are useful for virus classification (Koonin and Dolja 1993; Baker and Schroeder 2008). Here, we used them to estimate pollen-associated virus sharing between plant species, though they do also factor into the “relaxed” estimates of pollen-associated virus sharing (see below). A full list of viral entities obtained is available in Supplementary Tables S5–S7.

### Pollen-associated virus richness

We calculated “conservative” and “relaxed” estimates of pollen-associated virus richness following the procedures of Fetters et al. (2022). The conservative estimate was restricted to the known viruses, coding-complete novel viral genomes, and coding-complete novel viral variants. The relaxed estimate included the conservative number of viruses, plus those identified by their RdRp CDs from both novel partial viral genomes and variants (Koonin and Dolja 1993; Baker and Schroeder 2008). Preliminary analyses showed that virus richness was not affected by the number of flowers sampled per species (conservative and relaxed, both *r* < 0.14, *P* > 0.59), nor individuals sampled (conservative: *r* = −0.07, *P* = 0.79; relaxed: *r* = 0.06, *P* = 0.82). Nevertheless, to be conservative, we included the number of flowers sampled per species as a potential variable in the phylogenetically controlled model selection. It, however, was never included in the best models (see below; Supplementary Tables S8 and S9).

### Plant phylogeny and test of phylogenetic signal

A phylogeny of the 18 plants was constructed based upon the PhytoPhylo maximum likelihood megaphylogeny of vascular plants (Zanne et al. 2014; Qian and Jin 2016) using the R packages “ape” (Paradis and Schliep 2019) and “phytools” (Revell 2012). We tested for phylogenetic signals in independent and dependent variables using the Pagel’s λ method (Pagel 1999) using the “phylosig” function in the “phytools” R package (Revell 2012). A phylogenetic signal was considered present if Pagel’s λ was significantly above zero. As the relaxed estimate of pollen-associated virus richness, PC1, and PC2 all exhibited significant phylogenetic signals (all λ > 0.568, all *P* < 0.041), we used phylogenetically controlled linear model selection.

### Phylogenetically controlled linear model selection and modeling averaging

Generalized least squares models with all possible combinations of predictors were defined and then compared via Akaike Information Criterion corrected for small sample sizes (AICc) using the function “model.sel” in the R package “MuMIn” (Barton 2025). We averaged the set of models within AICc < 2 from the best model to produce the conditional average model (Grueber et al. 2011; Burnham and Anderson 2022) using the function “model.avg” in the “MuMIn” package (Barton 2025). All models for both estimates of virus richness are presented in Supplementary Tables S8 and S9.

### Estimate of pollen-associated virus sharing between plant species

We assessed whether the plant species shared pollen-associated viruses. We considered known viruses as shared when they were found in more than one plant species and considered novel viral genomes and variants as shared when their RdRps were 80–100% identical, or if their % identities were above the International Committee for the Taxonomy of Viruses (ICTV 2025) family-specific species demarcation threshold. To illustrate viral sharing, we composed a network of co-flowering plant species with “igraph” (Csárdi and Nepusz 2006) and “ggraph” (Pedersen 2024). Node size was scaled to reflect the relaxed estimate of pollen-associated viruses found in each plant species and colored by pollinator diversity. Edges were colored by the number of viruses shared between pairs of plant species. All statistical analyses were performed using R (v4.3.1).

## Results and discussion

### Pollen from co-flowering plant species hosts a diversity of viruses

The 18 plant species hosted from zero to three (conservative) and zero to 15 (relaxed) pollen-associated viruses. The two estimates of viral richness were positively correlated (r = 0.77, *P* < 0.001). In all, we found five known viruses, 18 coding-complete novel viral genomes/variants, and 131 partial novel viral genomes/variants. Of the latter groups, 115 are represented by RdRp sequences. The pollen-associated viral taxa represent 25 viral families, though some are too novel to be classified into an existing family (Supplementary Tables S5–S7). All five known viruses are known to infect crop plants, with two—*Alfalfa* and *Brome mosaic viruses*—belonging to a viral family (Bromoviridae) that has caused devastating disease in crops worldwide (ICTV 2025), indicating host range expansion.

Nearly all viral taxa that we identified that could be classified at least at the family level have RNA genomes (∼95%), although some belong to families with DNA genomes (Supplementary Tables S5–S7). Most of the viral taxa that we found belong to families whose viruses encode movement and/or coat proteins (Roossinck 2005) and/or have an acute lifestyle (i.e., cause symptomatic infections and move actively through plant cells and tissues), which allow viruses to infect hosts both vertically and horizontally (Hamelin et al. 2016). Viruses with these traits may be better equipped to exploit the pollen niche (Fetters and Ashman 2023) and are potentially shared between co-flowering plants in the same community via pollinators. Notable exceptions include the many novel viral taxa that belong to families that have a persistent lifestyle (i.e., cause asymptomatic infections and move passively via cell division from infected gametes following fertilization) (Roossinck 2010) and canonically infect hosts only vertically (e.g., Partitiviridae). However, detecting such viruses in association with pollen follows our previous work (Fetters et al. 2022). We found eight instances of Partitiviridae virus sharing between plant species (Supplementary Table S10), implying horizontal transmission to new hosts.

### Plant traits, and secondarily pollination generalism, drive pollen-associated virus richness

Five of the eight floral and pollen traits loaded substantially (i.e., > 20%) onto the two dominant PCs (Supplementary Table S2). PC1 explained 41% of the variation, with higher values reflecting restricted, closed flowers with longer tubes. PC2 explained 29% of the variation, with higher values reflecting multiple, bilateral flowers (Table 1).

**Fig. 1:**
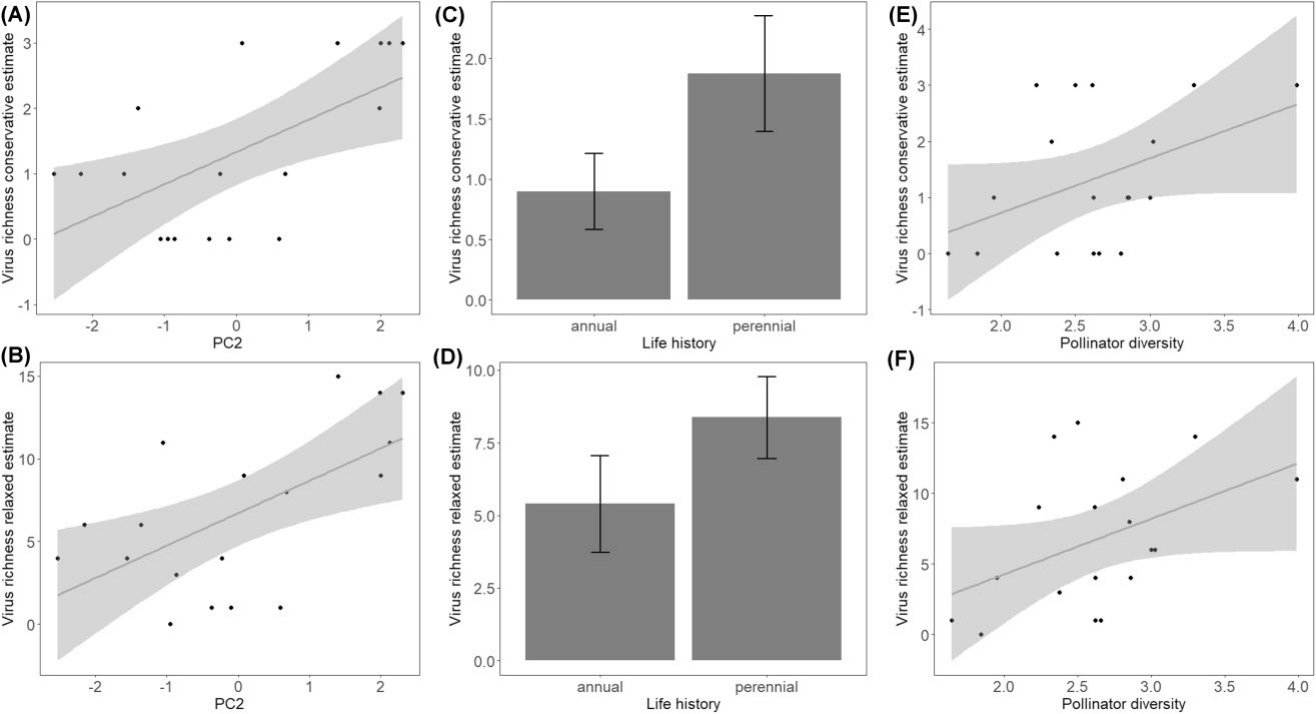
Plant trait principal component (PC) 2 (higher values reflecting multiple, bilateral flowers; A, B), life history (annual, perennial; C, D), and pollinator generalism (diversity of pollinators; E, F) associations with conservative and relaxed estimates of pollen-associated virus richness. Plants species are represented by dots and 95% confidence intervals by gray highlight (A, B, E, F). Error bars represent ± 1 standard error (C, D).

With respect to the conservative estimate of virus richness, models that included PC2, life history, and/or pollinator diversity received the best support (Supplementary Table S8). The averaged model showed that pollen from plants with higher values of PC2 (Fig. 1A; β_PC2_ ± adj. s.e.= 0.52 ± 0.12, z-value = 4.3, *P* < 0.001), perennial life history (Fig. 1C; β_LifeHistory_ ± adj. s.e.= 0.86 ± 0.43, z-value = 2.0, *P* = 0.046), and a greater diversity of pollinators (Fig. 1E; β_PollinatorDiversity_ ± adj. s.e = 0.83 + 0.43, z-value = 1.9, *P* = 0.05) had higher virus richness. Similarly, for the relaxed estimate, the best models included PC2, life history, and/or pollinator diversity (Supplementary Table S9). The averaged model demonstrated that plant species with higher values of PC2 (Fig. 1B; β_PC2_ ± adj. s.e = 1.9 ± 0.69, z-value = 2.7, *P* = 0.006), perennial life history (Fig. 1D; β_LifeHistory_ ± adj. s.e.= 2.6 ± 2.3, z-value = 1.1, *P* = 0.26), and pollinator diversity (Fig. 1F; β_PollinatorDiversity_ ± adj. s.e = 2.5 ± 2.0, z-value = 1.3, *P* = 0.21), had a greater virus richness, although the latter two were not statistically significant.

Of the floral traits that we assessed, bilateral floral symmetry and multiple-flowered inflorescences influence pollen-associated virus richness more than most, perhaps because they increase the likelihood a pollinator transfers infected pollen grains. Pollinators transfer more pollen (Minnaar et al. 2019; Stewart et al. 2022) and more epiphytic bacteria (Rebolleda-Gómez and Ashman 2019) when they interact closely with flowers, and the same could be true for pollen-associated viruses (Fetters and Ashman 2023). In addition, plant species with multiple-flowered inflorescences are likely to attract more pollinators than those with single flowers (e.g., Ohara and Higashi 1994; Koski et al. 2015), and plants with more flowers have been shown to also harbor more diverse fungal and bacterial (Wei et al. 2021b) and viral (Fetters et al. 2022) communities. That the same pattern with viral diversity was found across species from a continental scale (Fetters et al. 2022) suggests that traits that impact pollinator-plant contact at the level of organs within flower (bilateral symmetry) and flowers per plant (multi-flowered inflorescences) may be key to pollen-associated viral transfer. Life history was included in the best models, indicating a higher pollen viral richness for perennials than annuals. This result confirms that plants with multiple reproductive episodes and/or longer lifetimes are more likely to encounter, and build up viruses over time, potentially serving as reservoirs for pollen-associated viruses and those transmitted by other means, like herbivory (Ling et al. 2011).

We expected that a more attractive plant species, one visited by many species of pollinators, would harbor more diverse pollen-associated viruses, as suggested by theory for sexually transmitted diseases and mating system evolution (Hamilton 1990; Kokko et al. 2002; Wennström et al. 2003). Although we found that diversity of pollinators predicted the conservative estimate of pollen-associated virus richness, it was surprisingly less influential than floral morphology. It is possible that not all floral visitors forage for pollen or carry pollen or pollen-associated viruses, so plant species that are very attractive to pollinators, or are hubs within co-flowering communities, may not also be carrying numerous pollen-associated viruses. For example, Földesi et al. (2021) found that larger, wild bees deposited more pollen grains in single visits than smaller bees, honeybees, and flies, whereas our serpentine seep meta-community is heavily small bee- and fly-dominated (Wei et al. 2021a). Moreover, Zemenick et al. (2021) found that plants visited by many insect pollinators did not always host diverse communities of floral bacteria. Nevertheless, our results indicate that plant-pollinator interactions are associated with pollen viral diversity and strongly argue for future manipulative studies that would be able to disentangle the underlying mechanisms of transmission between (and within) plants, and separate pollination from non-pollination related mechanisms. For instance, one could exclude pollinators, or certain classes of pollinators, and assess the effects on pollen-associated virus richness. In addition, comparisons between flowers that are exposed to pollinators versus those that are never exposed to pollinators will distinguish between pollen viruses that are transmitted to the pollen from the mother plant (via past pollen-mediated infection) rather than via contemporary pollen-pollinator contact.

### Co-flowering plants share pollen-associated viruses

**Fig. 2:**
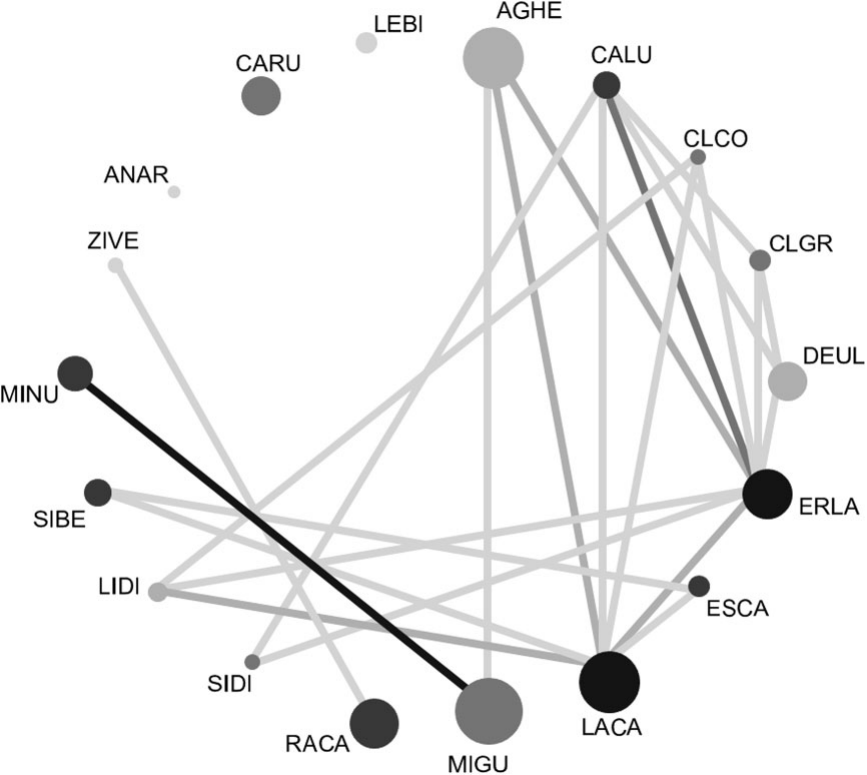
Network illustrating pollen-associated virus sharing among co-flowering plant species (nodes) in the community. Node size reflects the relaxed estimate of pollen-associated viruses, and node shading reflects a plant species’ pollinator generalism (darker = greater pollinator diversity). Lines connecting the nodes (edges) reflect shared viruses between connected species, and edge shading indicates the number of shared viruses between pairs of plant species (darker = more viruses shared). Unconnected species shared no viruses. Species codes as in Table 1.

We found that most of the co-flowering plant species shared one or more (up to 12) pollen-associated viruses with at least one other plant species in the community (Table 1, Supplementary Table S10). Two plant species—*Eriophyllum lanatum* and *Lasthenia californica*—appeared to be pollen-associated virus hubs in the community, as they shared 12 and 10 viruses with eight and seven other plant species, respectively (Fig. 2). In contrast, three plant species—*Anagallis arvensis, Castilleja rubicundula*, and *Leptosiphon bicolor—*hosted unique pollen-associated viruses, as they did not share these with any of the other focal species (Table 1; Fig. 2). Interestingly, across all plant species, the number of shared pollen-associated viruses was strongly positively correlated with pollinator diversity (*r* = 0.70, *P* = 0.004), as well as with both estimates of virus richness (both *r* > 0.56, *P* < 0.027).

Although flowering plants are known hubs of floral and bee microbes (e.g., Keller et al. 2021), including bee viruses (e.g., Singh et al. 2010), bee trypanosomatid pathogens (e.g., Figueroa et al. 2019), and bee-associated bacteria (e.g., McFrederick et al. 2017), this is the first study to show that not only do many co-flowering plant species share pollen-associated viruses, but that those with high pollinator diversity share the most. For instance, the most generalist species—*Eriophyllum lanatum* and *Lasthenia californica* (Table 1)—shared the most viruses with other plant species in the community (Fig. 2), whereas *Castilleja rubicundula, Anagallis arvensis*, and *Leptosiphon bicolor* shared no viruses with other plant species in the community. *Leptosphion bicolor* is a highly selfing species (Goodwillie et al. 2006), and the former two are among the plant species with the lowest levels of pollination generalism in the community (Table 1). It is hypothesized that pollination specialist plants may limit potential microbial transmission routes, as they are visited by only one or a few species of pollinator partners that may not visit other plant species, though this is not always the case (Keller et al. 2021). Moreover, the intriguing association with selfing suggests that future exploration should include explicit tests of the relationship between pollen virus diversity and plant mating system, and thus could directly test theory from sexually transmitted disease spread (Hamilton 1990; Kokko et al. 2002; Wennström et al. 2003). However, we note that the consequences of sharing the viral taxa we uncovered are not known, especially as the current study cannot determine whether or to what degree the viral taxa that we found are affecting their plant hosts. These viruses could be pathogenic (e.g., some Secoviridae; ICTV 2025), mutualistic (e.g., some Partitiviridae; Takahashi et al. 2019), or somewhere in between the two extremes (Roossinck 2015). Given the range of possible consequences of pollinator-mediated viral sharing, our study sets the stage for testing how sexually transmitted viruses affect plant fitness and whether they reflect sexually transmitted costs (disease) or benefits (e.g., mutualists; Moran and Dunbar 2006).

## Conclusion

This study uncovers for the first time the web of plant-virus interactions mediated by pollen transfer within a single co-flowering community. The association of pollen virus richness and traits that increase plant-pollinator interactions was confirmed and extended to include the roles of pollination generalism and life history. These novel associations beg greater research emphasis on pollinators as mediators of plant-viral interactions and the value of community-wide assessments on virus host range, and when paired with functional tests, will shed light on viral impacts on ecosystems.

## Author contributions

A.M.F., T.L.A., and J.M.P. designed the experiment; A.M.F., P.G.C., M.T.S.R., and T.L.A. collected the data; A.M.F., P.G.C., M.T.S.R., and T.L.A. analyzed the data; A.M.F. and T.L.A. produced the tables and figures and wrote the first draft of the manuscript; all authors contributed editorially to the manuscript, and author order was determined on the basis of these duties.

## Supplementary Material

icaf073_Supplemental_File

## Data and availability

The raw sequence reads are deposited in GenBank under Bioproject number PRJNA805217. Flower and pollen grain trait and pollinator diversity data were published in Wei et al. (2021a). All data related to this study and all R code generated for this study is available in Dryad (https://doi.org/10.5061/dryad.7wm37pw3k). In addition, any code for the Pickaxe pipeline is accessible in Github (https://github.com/pcantalupo/pickaxe).

## Notes

From the symposium “Pollinator-plant interactions in a changing landscape: embracing integrative approaches across scales” presented at the annual meeting of the Society for Integrative and Comparative Biology, January 3-7th, 2025.
