# Supplementary material for: Sharing Pollinators and Viruses: Virus Diversity of Pollen in a Co-Flowering Community
Source: Integr Comp Biol. 2025 Jun 2;65(4):942–54. doi: 10.1093/icb/icaf073 (PMC12530183; doi:10.1093/icb/icaf073)
Supplement: icaf073_Supplemental_File [file icaf073_supplemental_file.docx]

**Supplementary Table 1** The eight traits included in the principal component analysis (PCA), as well as plant species life history, their role in plant-pollinator interactions, and their hypothesized or demonstrated relationship to pollen-associated virus richness herein. If linkages are demonstrated to plant viruses in general, they are denoted by an asterisk; those that have demonstrated links to pollen-associated viruses are denoted with a ‘D’; and those only hypothesized with an ‘H’. Specific trait states predicted to increase a plant’s likelihood of attracting or interacting intimately or over repeated episodes with pollinators—the vectors of pollen-associated viruses—or having its pollen collected would lead to greater pollen-associated virus richness.

| **Trait** | **Role in plant-pollinator interactions** | **Trait state hypothesized or demonstrated to associate with increased pollen-associated virus richness** | **References** |
| --- | --- | --- | --- |
| Inflorescence type | Pollinator attraction | Multiple flowers | Fetters *et al.,* 2022^D^ |
| Flower size | Pollinator attraction | Larger | Fetters & Ashman 2023^H^ |
| Flower restrictiveness | Pollinator fit | Restrictive | Fetters & Ashman 2023^H^ |
| Flower shape | Pollinator fit | Closed | Fetters & Ashman 2023^H^ |
| Flower symmetry | Pollinator fit | Bilateral | Fetters *et al.,* 2022^D^ |
| Flower tube length | Pollinator fit | Longer | Fetters & Ashman 2023^H^ |
| Pollen grain texture | Pollen grain collectability | Spiky | Fetters *et al.,* 2022^D^ |
| Pollen grain length | Pollen grain collectability | Smaller | Fetters *et al.,* 2022^D^ |
| Life history | Number of reproductive episodes | Perennial | Fetters & Ashman 2023^H^; Ling *et al.,* 2011* |

**Supplementary Table 2** The percent contribution of the floral and pollen traits to the PCs from the PCA. Only those with a contribution of at least 20% (bold) were considered significant to a PC, and the first two PCs explained most of the variation (69%).

| **PC** | **Trait** | **Percent contribution** |
| --- | --- | --- |
| PC1 | **Flower restrictiveness** | **28.98** |
|  | **Flower tube length** | **27.70** |
|  | **Flower shape** | **24.87** |
|  | Pollen grain texture | 10.57 |
|  | Flower size | 4.92 |
|  | Pollen grain length | 2.18 |
|  | Flower symmetry | 0.75 |
|  | Inflorescence type | 0.02 |
| PC2 | **Flower symmetry** | **32.34** |
|  | **Inflorescence type** | **25.12** |
|  | Flower size | 16.29 |
|  | Pollen grain texture | 13.20 |
|  | Pollen grain length | 6.87 |
|  | Flower shape | 3.54 |
|  | Flower tube length | 2.30 |
|  | Flower restrictiveness | 0.33 |
| PC3 | **Pollen grain length** | **46.38** |
|  | **Flower size** | **20.27** |
|  | Pollen grain texture | 14.32 |
|  | Inflorescence type | 13.94 |
|  | Flower shape | 3.60 |
|  | Flower symmetry | 1.39 |
|  | Flower tube length | 0.08 |
|  | Flower restrictiveness | 0.002 |
| PC4 | **Inflorescence type** | **45.49** |
|  | Flower symmetry | 18.26 |
|  | Pollen grain length | 15.32 |
|  | Flower size | 12.90 |
|  | Pollen grain texture | 3.20 |
|  | Flower restrictiveness | 2.41 |
|  | Flower shape | 2.22 |
|  | Flower tube length | 0.20 |
| PC5 | **Flower symmetry** | **42.96** |
|  | **Flower size** | **32.55** |
|  | Pollen grain length | 8.22 |
|  | Pollen grain texture | 6.26 |
|  | Flower restrictiveness | 5.58 |
|  | Inflorescence type | 2.61 |
|  | Flower tube length | 0.95 |
|  | Flower shape | 0.87 |
| PC6 | **Pollen grain texture** | **51.85** |
|  | Flower size | 12.23 |
|  | Inflorescence type | 11.78 |
|  | Pollen grain length | 10.74 |
|  | Flower shape | 9.36 |
|  | Flower symmetry | 3.16 |
|  | Flower restrictiveness | 0.80 |
|  | Flower tube length | 0.08 |
| PC7 | **Flower tube length** | **64.22** |
|  | **Flower shape** | **26.26** |
|  | Flower restrictiveness | 4.18 |
|  | Pollen grain length | 3.47 |
|  | Flower symmetry | 1.12 |
|  | Pollen grain texture | 0.53 |
|  | Inflorescence type | 0.20 |
|  | Flower size | 0.02 |
| PC8 | **Flower restrictiveness** | **57.72** |
|  | **Flower shape** | **29.28** |
|  | Pollen grain length | 6.83 |
|  | Flower tube length | 4.46 |
|  | Inflorescence type | 0.83 |
|  | Flower size | 0.81 |
|  | Pollen grain texture | 0.07 |
|  | Flower symmetry | 0.003 |

**Supplementary Table 3** Sampling, total RNA extraction, total RNA quality check, total RNA sequencing, and Pickaxe information for each pollen sample.

| **Plant**  **spp^1^** | **GPS coordinates^2^** | **No. flowers^3^** | **No. plants^4^** | **Lysing (s)^5^** | **A260:**  **A280^6^** | **[Total RNA] (ng/ul)^7^** | **RIN^8^** | **No. raw reads^9^** | **No. non-plant reads^10^** | **No. VRS aligns^11^** | **No. QC contigs^12^** | **No. viral contigs^13^** |
| --- | --- | --- | --- | --- | --- | --- | --- | --- | --- | --- | --- | --- |
| *A. heterophylla* | 38.858489, -122.40941 | 18 | 18 | 105 | 2.12 | 59.0 | 7.6 | 205811288 | 141205960 | 599 | 10826 | 35 |
| *A. arvensis* | 38.866934, -122.452128 | 104 | 79 | 120 | 2.15 | 27.4 | 8.7 | 169310856 | 136531060 | 52 | 9420 | 3 |
| *C. luteus* | 38.862914, -122.399198 | 16 | 16 | 105 | 2.11 | 40.0 | 6.8 | 174344388 | 153202128 | 45 | 11053 | 16 |
| *C. rubicundula* | 38.857691, -122.408093 | 115 | 10 | 105 | 2.12 | 41.6 | 7.4 | 172185456 | 115560199 | 151 | 5783 | 20 |
| *C. concinna* | 38.862914, -122.399198 | 18 | 8 | 120 | 2.11 | 98.0 | 4.9 | 152896766 | 125444181 | 87 | 7635 | 1 |
| *C. gracilis* | 38.862914, -122.399198 | 5 | 5 | 120 | 2.12 | 100.0 | 5.1 | 152439328 | 125244257 | 1025 | 4421 | 7 |
| *D. uliginosum* | 38.859634, -122.411384 | 33 | 15 | 105 | 2.19 | 17.3 | 6.4 | 167365734 | 143458262 | 439 | 14852 | 25 |
| *E. lanatum* | 38.862914, -122.399198 | 70 | 12 | 105 | 2.13 | 32.9 | 7.7 | 133988080 | 93463702 | 448 | 7350 | 36 |
| *E. californica* | 38.857691, -122.408093 | 10 | 4 | 120 | 2.12 | 84.0 | 8.1 | 165676334 | 8112130 | 33 | 244 | 8 |
| *L. californica* | 38.857691, -122.408093 | 70 | 60 | 120 | 2.17 | 21.5 | 7.2 | 127276034 | 86371496 | 572 | 5572 | 58 |
| *L. bicolor* | 38.857691, -122.408093 | 145 | 41 | 120 | 2.20 | 26.0 | 7.4 | 187049058 | 10388268 | 21 | 2196 | 7 |
| *L. dichotomus* | 38.859634, -122.411384 | 38 | 38 | 105 | 2.10 | 74.0 | 4.2 | 184113090 | 151087762 | 101 | 12771 | 10 |
| *M. guttatus*  *(E. guttata)* | 38.857691, -122.408093 | 40 | 26 | 105 | 2.07 | 68.0 | 5.4 | 175153192 | 10502957 | 6111 | 217 | 31 |
| *M. nudatus*  *(E. nudata)* | 38.860515, -122.421054 | 116 | 110 | 105 | 2.20 | 20.2 | 8.1 | 173751624 | 11714333 | 194 | 420 | 14 |
| *R. californicus* | 38.867335, -122.451702 | 52 | 19 | 105 | 2.07 | 49.9 | 5.2 | 167983642 | 139854775 | 145 | 7237 | 34 |
| *S. diploscypha* | 38.867335, -122.451702 | 7 | 7 | 105 | 2.09 | 49.1 | 1.7 | 167468180 | 93146022 | 1895 | 2367 | 3 |
| *S. bellum* | 38.857691, -122.408093 | 45 | 20 | 105 | 2.06 | 89.0 | 7.4 | 162165480 | 138625878 | 352 | 9351 | 18 |
| *Z. venenosus*  *(T. venenosum)* | 38.866934, -122.452128 | 60 | 9 | 120 | 2.25 | 20.1 | 7.2 | 167495278 | 143954216 | 875 | 9009 | 5 |

^1-2^Plant spp, GPS coordinates: plant species and site from which a pollen sample was collected

^3-4^No. flowers, No. plants: number of flowers and individual plants from which a pollen sample was collected

^5^Lysing (s): number of seconds a pollen sample was disrupted using a Qiagen Tissue Lyser II

^6-8^A260:A280, [Total RNA] (ng/ul), RIN (RNA integrity number): purity ratio indicating level of degradation (higher numbers indicate less degradation), concentration of RNA extracted, and quality of extracted RNA as measured by a NanoDrop spectrophotometer, Qubit fluorometer, and TapeStation analysis at the GRC (University of Pittsburgh), respectively

^9^No. raw reads: total number of raw reads obtained from sequencing

^10^No. non-plant reads: number of reads that remained following the Pickaxe subtraction step

^11^No. VRS aligns: total number of times the non-plant reads aligned to VRS using Pickaxe

^12^No. QC (Quality control) contigs: number of contigs that remained following the Pickaxe contig assembly step and the steps that removed contigs that were too short, heavily masked contigs, or contained highly repetitive sequences

^13^No. viral contigs: number of viral contigs or extended contigs detected by Pickaxe

**Supplementary Table 4** Plant genomes included in each customized subtraction library.

| **Plant species^1^** | **Genomes included^2^** | **NCBI genome taxon no.^3^** |
| --- | --- | --- |
| *Agoseris heterophylla* | *Artemisia annua* L.  *Helianthus annuus* L.  *Lactuca sativa* L.  *Silybum marianum* (L.) Gaertn. | 2301  351  352  40483 |
| *Anagallis arvensis* | *Argania spinosa* (L.) Skeels  *Diospyros lotus* L.  *Embelia ribes* Burm. f.  *Primula veris* L.  *Primula vulgaris* Huds. | 70249  34476  46551  35300  38783 |
| *Calochortus luteus* | *Apostasia shenzhenica* Z. J. Liu & L. J. Chen  *Gastrodia elata* Blume  *Phalaenopsis equestris* (Schauer) Rchb. f.  *Phalaenopsis* hybrid cultivar | 66931  67401  11403  34687 |
| *Castilleja rubicundula* | *Mentha longifolia* (L.) Huds.  *Mimulus guttatus* Fisch. ex DC.  *Ocimum tenuiflorum* L.  *Perilla citriodora* (Makino) Nakai  *Pogostemon cablin* (Blanco) Benth.  *Salvia splendens* Ker Gawl.  *Scutellaria baicalensis* Georgi  *Utricularia gibba* L. | 44852  497  40058  46088  73046  77914  38543  16713 |
| *Clarkia concinna* | *Eucalyptus grandis* W. Hill ex Maiden  *Eugenia uniflora* L.  *Metrosideros polymorpha* Gaudich.  *Punica granatum* L. | 2181  16049  45178  13946 |
| *Clarkia gracilis* | *Eucalyptus melliodora* A. Cunn. ex Schauer  *Metrosideros polymorpha* Gaudich.  *Psidium guajava* L.  *Punica granatum* L. | 23986  45178  52475  13946 |
| *Delphinium uliginosum* | *Aquilegia coerulea* E. James  *Berberis thunbergii* DC. | 11153  15472 |
| *Eriophyllum lanatum* | *Artemisia annua* L.  *Chrysanthemum seticuspe* (Maxim.) Hand.-Mazz.  *Cynara cardunculus* L.  *Erigeron canadensis* L.  *Silybum marianum* (L.) Gaertn. | 2301  76498  11286  12828  40483 |
| *Eschscholzia californica* | *Eschscholzia californica* Cham.  *Macleaya cordata* (Willd.) R. Br.  *Papaver somniferum* L. | 12877  12912  12819 |
| *Lasthenia californica* | *Chrysanthemum seticuspe* (Maxim.) Hand.-Mazz.  *Erigeron canadensis* L.  *Helianthus annuus* L.  *Silphium perfoliatum* L.  *Silybum marianum* (L.) Gaertn. | 76498  12828  351  74235  40483 |
| *Leptosiphon bicolor*  *(Linanthus bicolor)* | *Argania spinosa* (L.) Skeels  *Diospyros lotus* L.  *Embelia ribes* Burm. f.  *Primula veris* L.  *Primula vulgaris* Huds. | 70249  34476  46551  35300  38783 |
| *Linanthus dichotomus* | *Actinidia chinensis* Planch.  *Argania spinosa* (L.) Skeels  *Diospyros lotus* L.  *Embelia ribes* Burm. f. | 16401  70249  34476  46551 |
| *Mimulus guttatus*  *(Erythranthe guttata)* | *Genlisea aurea* A. St. Hil  *Fraxinus excelsior* L  *Handroanthus impetiginosus* (Mart. Ex DC.) Mattos  *Mentha longifolia* (L.) Huds.  *Mimulus guttatus* Fisch. ex DC.  *Ruellia speciosa* (Mart. ex Nees)  *Sesamum indicum* L.  *Utricularia gibba* L. | 24580  31117  64326  44852  497  50955  11560  16713 |
| *Mimulus nudatus*  *(Erythranthe nudata)* | *Mentha longifolia* (L.) Huds.  *Mimulus guttatus* Fisch. ex DC.  *Ocimum tenuiflorum* L.  *Perilla citriodora* (Makino) Nakai  *Pogostemon cablin* (Blanco) Benth.  *Salvia splendens* Ker Gawl.  *Scutellaria baicalensis* Georgi  *Utricularia gibba* L. | 44852  497  40058  46088  73046  77914  38543  16713 |
| *Ranunculus californicus* | *Aquilegia coerulea* E. James  *Berberis thunbergii* DC. | 11153  15472 |
| *Sidalcea diploscypha* | *Corchorus capsularis* L.  *Corchorus olitorius* L.  *Durio zibethinus* L.  *Gossypium raimondii* Ulbr.  *Herrania umbratica* R. E. Schult.  *Hibiscus syriacus* L. | 46591  46639  57226  3239  55117  37069 |
| *Sisyrinchium bellum* | *Apostasia shenzhenica* Z. J. Liu & L. J. Chen  *Asparagus officinalis* L.  *Phalaenopsis* hybrid cultivar  *Vanilla planifolia* Andrews | 66931  10978  34687  17745 |
| *Zigadenus venenosus*  *(Toxicoscordion venenosum)* | *Dendrobium catenatum* Lindl.  *Gastrodia elata* Blume  *Phalaenopsis equestris* (Schauer) Rchb. f.  *Phalaenopsis* hybrid cultivar | 69090  67401  11403  34687 |

^1^Plant species: plant species from which a pollen sample was collected

^2^Genomes included: plant genomes included in the customized subtraction library for each pollen sample

^3^NCBI genome taxon no.: taxon number for each genome included in the customized subtraction library for each pollen sample

**Supplementary Table 5** Known viruses identified by read alignments to VRS. NCBI accession numbers indicate the top hit from the alignments to the VRS database.

| **Virus family^1^** | **Virus genus^2^** | **Known virus** | **Plant species^3^** | **No. segments recovered^4^** | **Percent sequence coverage^5^** | **No. alignments^6^** | **NCBI accession nos.** |
| --- | --- | --- | --- | --- | --- | --- | --- |
| *Bromoviridae* | *Alfamovirus* | Alfalfa mosaic virus | *Eriophyllum lanatum* | 3/3 | 46.41  46.90  53.95 | 63  40  51 | NC_001495.1 (RNA-1)  NC_002024.1 (RNA-2)  NC_002025.1 (RNA-3) |
|  | *Bromovirus* | Brome mosaic virus | *Lasthenia californica* | 3/3 | 38.90  62.16  64.47 | 54  72  60 | NC_002026.1 (RNA-1)  NC_002027.1 (RNA-2)  NC_002028.1 (RNA-3) |
| *Luteoviridae* | *Polerovirus* | Turnip yellows virus | *Lasthenia californica* | 1/1 | 32.28 | 66 | NC_003743.1 |
| *Partitiviridae* | *Betapartitivirus* | Red clover cryptic virus 2 | *Mimulus guttatus* | 1/2 | 20.45 | 1041 | NC_021096.1 (dsRNA1) |
|  | unclassified | Spinach cryptic virus 1 | *Agoseris heterophylla* | 1/2 | 21.72 | 136 | NC_033770.1 (dsRNA1) |
|  |  |  | *Mimulus guttatus* | 1/2 | 20.60 | 420 | NC_033770.1 (dsRNA1) |

^1-2^Virus family, genus: viral family and genus to which a known virus belongs

^3^Plant species: plant species in which a known virus was identified

^4^No. segments recovered: if the denominator is >1, a known virus has a segmented genome; the numerator denotes number of segments recovered

^5^Percent sequence coverage: the percentage of a top VRS hit covered by the reads; considered present if at least 20%

^6^No. alignments: the number of times the reads aligned to a top VRS hit; considered present if at least 10

**Supplementary Table 6** Novel coding-complete viral genomes and novel coding-complete variants identified. NCBI accession numbers are reflective of the top hit from either BLAST or RAPSearch2 search algorithms.

| Putative virus family^1^ | Putative virus name^2^ | Plant spp^3^ | No.  segs^4^ | CD identified^5^ | Length (nt)^6^ | Query  coverage^7^ | Rel.  abund.^8^ | Nt %  ID^9^ | FS % ID thresh^10^ | A^11^ | NCBI accession nos. |
| --- | --- | --- | --- | --- | --- | --- | --- | --- | --- | --- | --- |
| *Amalgaviridae* | Castilleja rubicundula amalgavirus 1 | *C. rubicundula* | 1 | RdRp | 3459 | 46.40 | 1.85 | 55.90 | 75%  [amino acid sequences] | r | AIX09819.1 |
|  | Castilleja rubicundula amalgavirus 2 |  |  |  | 3455 | 55.57 | 2.73 | 63.90 |  |  | YP_009388304.1 |
|  | Delphinium uliginosum amalgavirus 1 | *D. uliginosum* |  |  | 3461 | 62.24 | 1.18 | 57.10 |  |  | DAB41441.1 |
|  | Mimulus guttatus amalgavirus 1 | *M.*  *guttatus* |  |  | 3477 | 55.22 | 11.73 | 60.50 |  |  | YP_009388304.1 |
|  | Mimulus nudatus amalgavirus 1 | *M.*  *nudatus* |  |  | 3488 | 55.05 | 2.35 | 60.50 |  |  | YP_009388304.1 |
| *Betaflexiviridae* | Eriophyllum lanatum betaflexivirus 1 | *E.*  *lanatum* | 1 | methyltransferase/  helicase/  RdRp/movement/  coat | 6174 | 4.03 | 0.45 | 40.00 | 72% (coat or RdRp)  [nucleotide sequences] | r | ARQ83864.1 |
| *Endornaviridae* | Delphinium uliginosum endornavirus 1 | *D. uliginosum* | 1 | helicase/  glycosyltransferase | 11322 | 2.09 | 1.32 | 36.70 | 80%  [nucleotide sequences] | r | YP_009310116.1 |
|  | Leptosiphon bicolor endornavirus 1 | *L.*  *bicolor* |  | helicase/  capsular polysaccharide synthase/  RdRp | 16182 | 6.93 | 1.38 | 43.20 |  |  | YP_009222598.1 |
|  | Sisyrinchium bellum endornavirus 1 | *S.*  *bellum* |  | helicase/  methyltransferase/RdRp | 13542 | 8.93 | 97.37 | 52.40 |  |  | YP_009212849.1 |
| *Narnaviridae* | *Ocimum basilicum*  *RNA virus 2*  (novel variant 1) | *L.*  *californica* | 1 | RdRp | 2785 | 56.12 | 644.22 | 58.20 | 40 – 50%  [amino acid sequences] | r | YP_009408146.1 |
| *Nodaviridae* | Agoseris heterophylla nodavirus 1 | *A. heterophylla* | 2 | RdRp/RNA binding protein B2 (RNA1)  coat protein (RNA2) | 3133  1919 | 83.95  37.21 | 0.25  0.31 | 65.70  64.70 | 80% (coat) [nucleotide sequences] | r | AMO03244.1  ABB71128.1 |
| *Secoviridae* | Calochortus luteus secovirus 1 | *C.*  *luteus* | 2 | helicase/protease/  RdRp (RNA-1)  coat (RNA-2) | 7552  4320 | 7.31  20.69 | 1.26  2.18 | 53.90  36.70 | 80% (protease-RdRp) or 75% (coat)  [amino acid sequences] | r | AIT39627.1  AFB82732.1 |
|  | Clarkia gracilis secovirus 1 | *C.*  *gracilis* |  |  | 8313  6729 | 8.71  19.68 | 2.67  4.34 | 52.40  49.40 |  |  | AGR65698.1  AFB82732.1 |
|  | Delphinium uliginosum secovirus 1 | *D. uliginosum* |  |  | 6967  4861 | 44.28  22.22 | 1.83  3.28 | 54.80  49.40 |  |  | ANE06572.1  AFB82732.1 |
|  | Eriophyllum lanatum secovirus 1 | *E.*  *lanatum* |  |  | 7965  6373 | 8.73  8.88 | 2.79  3.11 | 52.90  29.10 |  |  | AGR65696.1  AFB82732.1 |
|  | Castilleja rubicundula secovirus 1 | *C. rubicundula* |  | helicase/RdRp (RNA-1)  coat (RNA-2) | 6539  7075 | 6.79  39.00 | 68.83  345.43 | 36.20  70.37 |  | r/t | CAJ33467.2  NC_015493.1 |
|  | Sisyrinchium bellum secovirus 1 | *S.*  *bellum* |  |  | 5590  4458 | 13.58  3.97 | 7.00  19.92 | 44.70  45.80 |  | r | AEN25475.1  NP_620620.2 |
| *Tombusviridae* | Eschscholzia californica tombusvirus 1 | *E. californica* | 1 | RdRp/coat | 3680 | 27.47 | 1.35 | 51.30 | 85%  [amino acid sequences] | r | AAT69238.1 |

^1^Putative virus family: family to which a novel coding-complete viral genome or variant belongs

^2^Putative virus name: novel coding-complete viral genomes were named after the plant species in which they were identified, as well as the putative viral families to which they belong; the novel variant is indicated beneath the name of the known virus

^3^Plant spp: plant species in which a novel coding-complete viral genome or variant was discovered

^4^No. segs (segments): the number of segments in a novel coding-complete viral genome or variant

^5^CD identified: the CDs bioinformatically identified in a novel coding-complete viral genome or variant

^6^Length (nt): length of a novel coding-complete viral genome or variant

^7^Query coverage: the percent of a novel coding-complete viral genome or variant that participated in the alignment with the top BLAST or RAPSearch2 hit

^8^Rel. (Relative) abund. (abundance): the number of reads assembled into a novel coding-complete viral genome or variant, divided by its genome length

^9^Nt % ID: similarity of a novel coding-complete viral genome or variant to the top BLAST or RAPSearch2 hit, where the two align

^10^FS (Family-specific) % ID thresh (threshold): novelty assigned based upon ICTV percentage identity criteria for nucleotide or amino acid sequences or specific CDs

^11^A (Algorithm): search algorithm used to find similarity between a novel coding-complete viral genome or variant and NCBI nucleotide or protein databases; r = rapsearch, t = tblastx

**Supplementary Table 7** Novel partial viral genomes and novel partial variants of known viruses identified. NCBI accession numbers are reflective of the top hit from either BLAST or Rapsearch2 search algorithms. Bolded putative virus names indicate the novel partial viral genomes or variants in which a RdRp CD was identified and therefore included in the relaxed estimate of virus richness for the plant species in which they were discovered and in the estimation of pollen-associated virus sharing.

| **Putative virus family^1^** | **Putative**  **virus name^2^** | **Plant species^3^** | **CD identified^4^** | **Length (nt)^5^** | **Query**  **cover^6^** | **Rel.**  **abund.^7^** | **Nt %**  **ID^8^** | **FS % ID thresh^9^** | **A^10^** | **NCBI accession nos.** |
| --- | --- | --- | --- | --- | --- | --- | --- | --- | --- | --- |
| *Alphaflexiviridae* | Linanthus dichotomus  alphaflexivirus 1 | *Linanthus dichotomus* | nucleic acid binding protein | 1032 | 35.17 | 0.48 | 33.10 | 72% (coat or RdRp)  [nucleotide sequences] | r | AHA31805.1 |
| *Amalgaviridae* | **Delphinium uliginosum**  **amalgavirus 2** | *Delphinium uliginosum* | RdRp | 2184 | 98.43 | 0.45 | 61.50 | 75%  [amino acid sequences] | r | DAB41439.1 |
|  | **Linanthus dichotomus**  **amalgavirus 1** | *Linanthus dichotomus* | RdRp | 1590 | 36.04 | 0.24 | 49.20 |  |  | DAB41441.1 |
|  | **Ranunculus californicus**  **amalgavirus 1** | *Ranunculus californicus* | RdRp | 2978 | 100.00 | 0.42 | 67.50 |  |  | YP_003934623.1 |
|  | **Ranunculus californicus**  **amalgavirus 2** |  | RdRp | 2945 | 98.15 | 0.85 | 64.80 |  |  | DAB41439.1 |
|  | **Sisyrinchium bellum**  **amalgavirus 1** | *Sisyrinchium bellum* | RdRp | 3039 | 99.62 | 0.86 | 54.90 |  |  | DAB41439.1 |
| *Benyviridae* | **Mimulus guttatus**  **benyvirus 1** | *Mimulus guttatus* | helicase/  RdRp | 6089 | 4.43 | 2.82 | 38.50 | 60%  [amino acid sequences] | r | ABU94739.2 |
|  | **Mimulus nudatus**  **benyvirus 1** | *Mimulus nudatus* | helicase/RdRp | 8550 | 3.12 | 0.52 | 31.50 |  |  | NP_612615.1 |
| *Betaflexiviridae* | Anagallis arvensis  betaflexivirus 1 | *Anagallis arvensis* | nucleic acid binding protein | 884 | 33.00 | 0.77 | 37.88 | 72% (coat or RdRp)  [nucleotide sequences] | t | NC_002500.1 |
|  | Eriophyllum lanatum  betaflexivirus 2 | *Eriophyllum lanatum* | methyltransferase | 659 | 98.33 | 0.16 | 65.30 |  | r | AQQ73540.1 |
|  | Lasthenia californica  betaflexivirus 1 | *Lasthenia californica* | coat | 812 | 43.60 | 0.23 | 55.10 |  |  | CDW92035.1 |
|  | ***Apple stem grooving virus***  **(novel variant 1)** | *Ranunculus californicus* | RdRp | 537 | 98.88 | 0.17 | 76.80 |  |  | APT42870.1 |
|  | **Zigadenus venenosus**  **betaflexivirus 1** | *Zigadenus venenosus* | RdRp/  movement/coat | 2339 | 68.29 | 0.33 | 56.10 |  |  | BBA57167.1 |
|  | Zigadenus venenosus  betaflexivirus 2 |  | unknown function | 1273 | 27.81 | 0.48 | 41.50 |  |  | ALF38090.1 |
|  | Zigadenus venenosus  betaflexivirus 3 |  | methyltransferase | 548 | 78.83 | 0.36 | 47.20 |  |  | ACD88337.1 |
| *Bromoviridae* | ***Grapevine virus S***  **(novel variant 1)** | *Delphinium uliginosum* | RdRp | 2868 | 72.00 | 0.83 | 99.47 | 80%  [nucleotide sequences] | tb | JX513899.1 |
|  | Delphinium uliginosum  bromovirus 1 |  | methyltransferase/helicase | 3474 | 48.70 | 0.84 | 78.40 |  | r | AQS99321.2 |
|  | Delphinium uliginosum  bromovirus 2 |  | movement/coat | 2236 | 37.16 | 1.28 | 70.90 |  |  | ANN11740.1 |
| *Caulimoviridae* | Delphinium uliginosum  caulimovirus 1 | *Delphinium uliginosum* | reverse transcriptase/  DNA binding protein | 3408 | 14.79 | 1.95 | 58.30 | 80%  [nucleotide sequences] | r | AGQ49469.1 |
|  | Delphinium uliginosum  caulimovirus 2 |  | viroplasmin | 1185 | 18.73 | 1.16 | 79.70 |  |  | AEA39176.1 |
| *Chrysoviridae* | **Castilleja rubicundula**  **chrysovirus 1** | *Castilleja rubicundula* | RdRp | 1253 | 99.84 | 0.77 | 65.20 | 80%  [nucleotide sequences] | r | AKU48197.1 |
| *Dicistroviridae* | Eriophyllum lanatum  dicistrovirus 1 | *Eriophyllum lanatum* | coat | 1456 | 61.00 | 0.16 | 27.42 | 80%  [nucleotide sequences] | t | NC_035184.1 |
|  | Eriophyllum lanatum  dicistrovirus 2 |  | helicase | 1076 | 27.88 | 0.18 | 45.00 |  | r | ANS71513.1 |
|  | **Eriophyllum lanatum**  **dicistrovirus 3** |  | RdRp | 1002 | 45.51 | 0.14 | 37.60 |  |  | ASM93982.1 |
| *Endornaviridae* | **Delphinium uliginosum**  **endornavirus 2** | *Delphinium uliginosum* | RdRp | 3338 | 16.15 | 0.86 | 34.90 | 80%  [nucleotide sequences] | r | AQM32768.1 |
|  | **Delphinium uliginosum**  **endornavirus 3** |  | RdRp | 2330 | 33.76 | 1.49 | 34.30 |  |  | AQM32768.1 |
|  | Delphinium uliginosum  endornavirus 4 |  | helicase | 1895 | 9.34 | 0.83 | 51.60 |  |  | AQM32768.1 |
| *Geminiviridae* | *Tomato yellow leaf curl Indonesia virus*  (novel variant 1) | *Linanthus dichotomus* | replication protein | 754 | 19.72 | 0.39 | 78.40 | 75%  [nucleotide sequences] | r | YP_699993.1 |
| *Mayoviridae* | Sidalcea diploscypha  mayovirus 1 | *Sidalcea diploscypha* | coat | 1467 | 21.00 | 0.54 | 51.61 | 80%  [nucleotide sequences] | t | NC_034390.1 |
| *Narnaviridae* | ***Ocimum basilicum RNA virus 2***  **(novel variant 2)** | *Clarkia concinna* | RdRp | 945 | 99.37 | 0.15 | 58.80 | 40 – 50%  [amino acid sequences] | r | YP_009408146.1 |
|  | ***Ocimum basilicum RNA virus 2***  **(novel variant 3)** | *Eriophyllum lanatum* | RdRp | 542 | 99.08 | 0.12 | 72.10 |  |  | YP_009408146.1 |
|  | ***Cronartium ribicola mitovirus 2***  **(novel variant 1)** | *Eschscholzia californica* | RdRp | 1039 | 45.21 | 0.18 | 52.90 |  |  | YP_009259481.1 |
|  | ***Ocimum basilicum RNA virus 2***  **(novel variant 4)** | *Linanthus dichotomus* | RdRp | 1436 | 64.74 | 1.44 | 58.80 |  |  | YP_009408146.1 |
|  | ***Ocimum basilicum RNA virus 2***  **(novel variant 5)** |  | RdRp | 1214 | 91.19 | 1.96 | 58.30 |  |  | YP_009408146.1 |
| No family | **Agoseris heterophylla**  **no family 1** | *Agoseris heterophylla* | RdRp | 7914 | 1.58 | 0.54 | 28.20 | 80%  [nucleotide sequences] | r | YP_009336924.1 |
|  | **Agoseris heterophylla**  **no family 2** |  | RdRp | 3812 | 6.14 | 0.19 | 41.70 |  |  | APG79349.1 |
|  | **Agoseris heterophylla**  **no family 3** |  | RdRp/RNA binding protein B2 | 3133 | 90.30 | 0.17 | 62.20 |  |  | APG76332.1 |
|  | **Agoseris heterophylla**  **no family 4** |  | RdRp | 1846 | 86.23 | 0.15 | 54.60 |  |  | YP_009337870.1 |
|  | **Agoseris heterophylla**  **no family 5** |  | RdRp | 547 | 46.62 | 0.08 | 46.10 |  |  | YP_009342285.1 |
|  | **Calochortus luteus**  **no family 1** | *Calochortus luteus* | RdRp | 2664 | 84.51 | 0.35 | 63.20 |  |  | YP_009337376.1 |
|  | Calochortus luteus  no family 2 |  | coat | 1561 | 24.98 | 0.74 | 36.40 |  |  | APG75767.1 |
|  | **Castilleja rubicundula**  **no family 1** | *Castilleja rubicundula* | RdRp | 9125 | 0.46 | 9.71 | 35.70 |  |  | YP_009182153.1 |
|  | **Clarkia gracilis**  **no family 1** | *Clarkia gracilis* | RdRp | 1080 | 2.46 | 0.37 | 71.40 |  |  | YP_009336820.1 |
|  | **Delphinium uliginosum**  **no family 1** | *Delphinium uliginosum* | RdRp | 2974 | 20.48 | 1.54 | 37.90 |  |  | YP_009342285.1 |
|  | Delphinium uliginosum  no family 2 |  | methyltransferase | 2462 | 17.00 | 2.12 | 33.81 |  | t | NC_033436.1 |
|  | Eriophyllum lanatum  no family 1 | *Eriophyllum lanatum* | protease/nucleic acid binding protein | 1220 | 34.00 | 0.20 | 32.90 |  |  | NC_032766.1 |
|  | Eriophyllum lanatum  no family 2 |  | coat | 847 | 41.79 | 0.18 | 38.00 |  | r | YP_009272816.1 |
|  | **Eriophyllum lanatum**  **no family 3** |  | RdRp | 587 | 91.48 | 0.10 | 43.90 |  |  | APG78016.1 |
|  | **Lasthenia californica**  **no family 1** | *Lasthenia californica* | coat/helicase/  RdRp | 9027 | 4.69 | 2.45 | 34.50 |  |  | AEM65163.1 |
|  | **Lasthenia californica**  **no family 2** |  | coat/helicase/  protease/RdRp | 8867 | 12.99 | 0.47 | 34.20 |  |  | APG78623.1 |
|  | **Lasthenia californica**  **no family 3** |  | helicase/RdRp | 6436 | 7.36 | 2.56 | 47.80 |  |  | YP_009336557.1 |
|  | Lasthenia californica  no family 4 |  | coat | 4020 | 0.97 | 0.81 | 53.80 |  |  | ASH89122.1 |
|  | **Lasthenia californica**  **no family 5** |  | RdRp | 2211 | 70.00 | 0.14 | 40.50 |  |  | APG79256.1 |
|  | Lasthenia californica  no family 6 |  | coat | 1664 | 57.82 | 2.31 | 38.20 |  |  | APG76763.1 |
|  | **Lasthenia californica**  **no family 7** |  | RdRp | 700 | 93.43 | 0.12 | 33.90 |  |  | APG79293.1 |
|  | **Leptosiphon bicolor**  **no family 1** | *Leptosiphon bicolor* | RdRp | 1860 | 85.32 | 1.31 | 48.70 |  |  | YP_009337870.1 |
|  | **Leptosiphon bicolor**  **no family 2** |  | RdRp | 1496 | 99.51 | 0.07 | 69.50 |  |  | YP_009130618.1 |
|  | **Mimulus guttatus**  **no family 1** | *Mimulus guttatus* | RdRp | 9035 | 4.18 | 1.42 | 37.80 |  |  | YP_009182153.1 |
|  | **Mimulus guttatus**  **no family 2** |  | RdRp | 3513 | 10.65 | 1.11 | 37.80 |  |  | AGW51765.1 |
|  | **Mimulus guttatus**  **no family 3** |  | RdRp | 2053 | 24.55 | 11.53 | 31.80 |  |  | APG76021.1 |
|  | **Mimulus guttatus**  **no family 4** |  | RdRp | 1537 | 92.52 | 16.71 | 74.30 |  |  | YP_009026407.1 |
|  | **Mimulus nudatus**  **no family 1** | *Mimulus nudatus* | RdRp | 2710 | 10.27 | 4.93 | 21.20 |  |  | APG79216.1 |
|  | **Mimulus nudatus**  **no family 2** |  | RdRp | 2610 | 11.15 | 2.36 | 56.70 |  |  | YP_009182153.1 |
|  | **Mimulus nudatus**  **no family 3** |  | RdRp | 1784 | 4.37 | 2.33 | 46.20 |  |  | ALD89106.2 |
|  | **Mimulus nudatus**  **no family 4** |  | RdRp | 1253 | 53.33 | 0.15 | 31.80 |  |  | APG76021.1 |
|  | **Ranunculus californicus**  **no family 1** | *Ranunculus californicus* | RdRp | 805 | 99.88 | 0.19 | 64.90 |  |  | YP_009272911.1 |
| *Nudiviridae* | Lasthenia californica  nudivirus 1 | *Lasthenia californica* | DNA polymerase | 1355 | 56.01 | 0.27 | 61.80 | 80%  [nucleotide sequences] | r | YP_009345924.1 |
|  | Lasthenia californica  nudivirus 2 |  | Per os infectivity factor 2 | 586 | 99.83 | 0.22 | 64.00 |  |  | AKH40343.1 |
| *Partitiviridae* | **Agoseris heterophylla**  **partitivirus 1** | *Agoseris heterophylla* | RdRp | 2463 | 89.40 | 2.16 | 74.10 | 90% (RdRp) or 80% (coat)  [amino acid sequences] | r | YP_009293586.1 |
|  | **Agoseris heterophylla**  **partitivirus 2** |  | RdRp | 1988 | 98.00 | 1.14 | 81.11 |  | b | KX784754.1 |
|  | **Agoseris heterophylla**  **partitivirus 3** |  | RdRp | 1916 | 21.45 | 0.30 | 76.60 |  | r | ANQ45203.1 |
|  | **Agoseris heterophylla**  **partitivirus 4** |  | RdRp | 1624 | 87.19 | 51.00 | 60.70 |  |  | AAB27624.1 |
|  | **Agoseris heterophylla**  **partitivirus 5** |  | RdRp | 1391 | 99.83 | 0.17 | 69.80 |  |  | YP_001686783.1 |
|  | **Agoseris heterophylla**  **partitivirus 6** |  | RdRp | 1245 | 89.88 | 0.21 | 62.20 |  |  | ARO72610.1 |
|  | **Calochortus luteus**  **partitivirus 1** | *Calochortus luteus* | RdRp | 1627 | 84.82 | 4.21 | 55.50 |  |  | ARO72610.1 |
|  | **Calochortus luteus**  **partitivirus 2** |  | RdRp | 1128 | 99.20 | 0.42 | 82.80 |  |  | YP_007889821.1 |
|  | **Castilleja rubicundula**  **partitivirus 1** | *Castilleja rubicundula* | RdRp | 2022 | 91.39 | 1.25 | 81.30 |  |  | YP_008719882.1 |
|  | **Castilleja rubicundula**  **partitivirus 2** |  | RdRp | 1659 | 86.08 | 23.88 | 58.60 |  |  | ASU87378.1 |
|  | **Castilleja rubicundula**  **partitivirus 3** |  | RdRp | 1623 | 86.51 | 6.44 | 67.70 |  |  | APT68925.1 |
|  | **Clarkia gracilis**  **partitivirus 1** | *Clarkia gracilis* | RdRp | 2451 | 90.94 | 112.70 | 72.80 |  |  | YP_007889821.1 |
|  | **Clarkia gracilis**  **partitivirus 2** |  | RdRp | 1918 | 72.89 | 5.86 | 53.20 |  |  | AOR51388.1 |
|  | **Delphinium uliginosum**  **partitivirus 1** | *Delphinium uliginosum* | RdRp | 1745 | 80.63 | 11.34 | 66.30 |  |  | YP_004429258.1 |
|  | **Eriophyllum lanatum**  **partitivirus 1** | *Eriophyllum lanatum* | RdRp | 1687 | 84.29 | 0.29 | 66.50 |  |  | YP_001686783.1 |
|  | **Eriophyllum lanatum**  **partitivirus 2** |  | RdRp | 1681 | 83.88 | 0.47 | 60.90 |  |  | ARO72610.1 |
|  | **Eriophyllum lanatum**  **partitivirus 3** |  | RdRp | 1597 | 94.68 | 0.31 | 81.00 |  |  | YP_007889821.1 |
|  | **Eriophyllum lanatum**  **partitivirus 4** |  | RdRp | 1302 | 31.57 | 3.52 | 76.60 |  |  | BBA66577.1 |
|  | **Eschscholzia californica**  **partitivirus 1** | *Eschscholzia californica* | RdRp | 1356 | 25.00 | 0.39 | 50.40 |  |  | BBA66577.1 |
|  | **Lasthenia californica**  **partitivirus 1** | *Lasthenia californica* | RdRp | 1832 | 18.50 | 0.26 | 50.40 |  |  | BBA66577.1 |
|  | **Lasthenia californica**  **partitivirus 2** |  | RdRp | 680 | 99.71 | 0.18 | 53.50 |  |  | AOR51388.1 |
|  | **Leptosiphon bicolor**  **partitivirus 1** | *Leptosiphon bicolor* | RdRp | 1919 | 8.44 | 0.36 | 38.10 |  |  | ANQ45203.1 |
|  | **Mimulus guttatus**  **partitivirus 1** | *Mimulus guttatus* | RdRp | 2413 | 92.75 | 22.49 | 77.20 |  |  | YP_007889825.1 |
|  | **Mimulus guttatus**  **partitivirus 2** |  | RdRp | 1963 | 98.00 | 3.14 | 80.07 |  | b | FJ550604.1 |
|  | **Mimulus guttatus**  **partitivirus 3** |  | RdRp | 1624 | 86.45 | 128.46 | 67.70 |  | r | APT68925.1 |
|  | **Mimulus nudatus**  **partitivirus 1** | *Mimulus nudatus* | RdRp | 1824 | 95.07 | 0.93 | 82.50 |  |  | YP_007889823.1 |
|  | ***Grapevine cryptic virus***  **(novel variant 1)** | *Ranunculus californicus* | RdRp | 1902 | 63.00 | 0.79 | 88.84 |  | b | JX658568.1 |
|  | **Ranunculus californicus**  **partitivirus 1** |  | RdRp | 1993 | 90.17 | 3.44 | 69.20 |  | r | AJE25830.1 |
|  | **Ranunculus californicus**  **partitivirus 2** |  | RdRp | 1989 | 87.93 | 1.25 | 73.60 |  |  | AFX73022.1 |
|  | **Ranunculus californicus**  **partitivirus 3** |  | RdRp | 1949 | 9.04 | 0.74 | 63.80 |  |  | BAQ36631.1 |
|  | **Ranunculus californicus**  **partitivirus 4** |  | RdRp | 1927 | 7.16 | 0.52 | 56.50 |  |  | AFX73022.1 |
|  | **Ranunculus californicus**  **partitivirus 5** |  | RdRp | 1897 | 82.71 | 0.93 | 64.20 |  |  | AOX47571.1 |
|  | **Ranunculus californicus**  **partitivirus 6** |  | RdRp | 728 | 98.90 | 1.06 | 76.70 |  |  | AOX47571.1 |
|  | **Sidalcea diploscypha**  **partitivirus 1** | *Sidalcea diploscypha* | RdRp | 2435 | 91.79 | 71.76 | 72.50 |  |  | YP_007891054.1 |
|  | **Sisyrinchium bellum**  **partitivirus 1** | *Sisyrinchium bellum* | RdRp | 2004 | 92.22 | 1.93 | 85.40 |  |  | ACL93278.1 |
|  | **Sisyrinchium bellum**  **partitivirus 2** |  | RdRp | 1697 | 19.98 | 0.38 | 49.60 |  |  | ANQ45203.1 |
|  | **Sisyrinchium bellum**  **partitivirus 3** |  | RdRp | 1547 | 97.97 | 40.38 | 74.30 |  |  | BBA57905.1 |
| *Peribunyaviridae* | **Castilleja rubicundula**  **peribunyavirus 1** | *Castilleja rubicundula* | RdRp | 4098 | 12.30 | 0.56 | 28.00 | 90%  [amino acid sequences] | r | ACV95628.1 |
|  | **Mimulus guttatus**  **peribunyavirus 1** | *Mimulus guttatus* | RdRp | 6615 | 2.72 | 2.96 | 41.70 |  |  | AJG39269.1 |
|  | **Mimulus guttatus**  **peribunyavirus 2** |  | RdRp | 6485 | 3.10 | 7.47 | 38.80 |  |  | AJG39269.1 |
|  | **Mimulus guttatus**  **peribunyavirus 3** |  | RdRp | 6414 | 3.13 | 13.90 | 40.30 |  |  | AJG39269.1 |
|  | **Mimulus guttatus**  **peribunyavirus 4** |  | RdRp | 4179 | 14.00 | 0.42 | 35.14 |  | t | NC_034459.1 |
| *Phenuiviridae* | **Agoseris heterophylla**  **phenuivirus 1** | *Agoseris heterophylla* | RdRp | 3812 | 10.00 | 0.18 | 35.20 | 80%  [nucleotide sequences] | r | AJG39254.1 |
|  | **Lasthenia californica**  **phenuivirus 1** | *Lasthenia californica* | RdRp | 2015 | 15.93 | 0.23 | 30.80 |  |  | AJG39235.1 |
|  | **Lasthenia californica**  **phenuivirus 2** |  | RdRp | 1507 | 47.45 | 0.18 | 32.00 |  |  | AJG39234.1 |
|  | **Lasthenia californica**  **phenuivirus 3** |  | RdRp | 1142 | 53.33 | 0.30 | 31.70 |  |  | AFN73042.1 |
|  | **Mimulus nudatus**  **phenuivirus 1** | *Mimulus nudatus* | RdRp | 637 | 50.00 | 5.95 | 26.17 |  | t | NC_032282.1 |
| *Phycodnaviridae* | *Phaeocystis globose virus*  (novel variant 1) | *Linanthus dichotomus* | viroplasmin | 1389 | 9.00 | 0.64 | 54.76 | 29 – 98% [nucleotide sequences] | t | NC_021312.1 |
| *Potyviridae* | **Calochortus luteus**  **potyvirus 1** | *Calochortus luteus* | RdRp | 1475 | 26.44 | 1.66 | 33.60 | 76% (entire genome) [nucleotide sequences],  82% (entire genome)  [amino acid sequences],  58% (protease) [nucleotide sequences],  74 – 78% (all other CDs)  [nucleotide sequences) | r | CAA63099.2 |
| *Retroviridae* | *Citrus endogenous pararetrovirus* (novel variant 1) | *Anagallis arvensis* | movement | 1328 | 63.84 | 0.26 | 61.70 | 50%  [nucleotide sequences] | r | YP_008992013.1 |
| *Secoviridae* | Agoseris heterophylla  secovirus 1 | *Agoseris heterophylla* | coat | 914 | 41.00 | 0.11 | 31.67 | 80% (protease-RdRp) or 75% (coat)  [amino acid sequences] | t | NC_034215.1 |
|  | Calochortus luteus  secovirus 2 | *Calochortus luteus* | coat | 4159 | 8.38 | 0.40 | 20.00 |  | r | AAO52686.1 |
|  | **Calochortus luteus**  **secovirus 3** |  | RdRp | 1728 | 10.57 | 0.25 | 62.50 |  |  | ABM65095.1 |
|  | **Eriophyllum lanatum**  **secovirus 2** | *Eriophyllum lanatum* | RdRp | 4022 | 2.83 | 0.86 | 68.40 |  |  | ABM65095.1 |
|  | Eriophyllum lanatum  secovirus 3 |  | helicase | 3402 | 28.40 | 0.79 | 39.40 |  |  | AGY34703.1 |
|  | Eriophyllum lanatum  secovirus 4 |  | coat | 1485 | 12.73 | 1.42 | 36.50 |  |  | ABL84276.1 |
|  | **Lasthenia californica**  **secovirus 1** | *Lasthenia californica* | helicase/RdRp | 8262 | 11.01 | 0.51 | 47.40 |  |  | AQW44800.1 |
|  | Lasthenia californica  secovirus 2 |  | coat | 3158 | 5.98 | 0.73 | 36.50 |  |  | ABL84276.1 |
| *Totiviridae* | **Eschscholzia californica**  **totivirus 1** | *Eschscholzia californica* | RdRp | 589 | 66.21 | 0.11 | 48.50 | 50 – 60%  [amino acid sequences] | r | ATO91011.1 |
|  | Sisyrinchium bellum  totivirus 1 | *Sisyrinchium bellum* | coat | 1438 | 43.18 | 0.71 | 44.00 |  |  | AOX47552.1 |
|  | Sisyrinchium bellum  totivirus 2 |  | coat | 1437 | 38.20 | 1.63 | 54.10 |  |  | AOX47552.1 |
| *Virgaviridae* | Delphinium uliginosum  virgavirus 1 | *Delphinium uliginosum* | helicase/  movement/coat | 2785 | 38.24 | 1.82 | 55.60 | 80%  [nucleotide sequences] | r | CAA86470.1 |
|  | **Delphinium uliginosum**  **virgavirus 2** |  | RdRp | 2397 | 67.46 | 1.05 | 78.50 |  |  | AIT18340.1 |
|  | Delphinium uliginosum  virgavirus 3 |  | helicase | 1819 | 80.32 | 0.36 | 60.40 |  |  | AAA79146.1 |

^1^Putative virus family: family to which a novel partial viral genome or variant belongs

^2^Putative virus name: novel partial viral genomes were named after the plant species in which they were identified, as well as the putative families to which they belong; where applicable, the numbering scheme is continued from Supplementary Table 5; novel variants are indicated beneath the names of the known viruses

^3^Plant species: plant species in which a novel partial viral genome or variant was discovered

^4^CD identified: the CDs bioinformatically identified in a novel partial viral genome or variant

^5^Length (nt): length of a novel partial viral genome or variant

^6^Query cover (coverage): the percent of a novel partial viral genome or variant that participated in the alignment with the top BLAST or RAPSearch2 hit

^7^Rel. (Relative) abund. (abundance): the number of reads assembled into a novel partial viral genome or variant, divided by its genome length

^8^Nt (Nucleotide) % ID: similarity of a novel partial viral genome or variant to the top BLAST or RAPSearch2 hit, where the two align

^9^FS (family-specific) % ID thresh (threshold): novelty assigned based upon ICTV percentage identity criteria for nucleotide or amino acid sequences or specific CDs

^10^A (Algorithm): search algorithm used to find similarity between a novel partial viral genome or variant and NCBI nucleotide or protein databases; b = blastn, t = tblastx, r = rapsearch

**Supplementary Table 8** Results of phylogenetically controlled model selection for the conservative estimate of pollen virome richness. Models are ranked by AICc value. Asterisks denote models of equal support that were averaged.

| **Model** | **(Intercept)** | **Number of sampled flowers** | **PC1** | **PC2** | **Pollinator diversity** | **Life history** | **df** | **logLik** | **AICc** | **delta** | **weight** |
| --- | --- | --- | --- | --- | --- | --- | --- | --- | --- | --- | --- |
| **Conditional average** | **0.5430** | **-** | **-** | **0.5190** | **0.8265** | **0.8582** | - | - | - | - | - |
| 3* | 1.2640 | **-** | **-** | 0.5587 | **-** | - | 4 | -25.409 | 61.9 | 0.00 | 0.308 |
| 14* | 0.9567 | - | - | 0.5397 | - | 0.8582 | 5 | -23.655 | 62.3 | 0.41 | 0.250 |
| 13* | -0.8703 | - | - | 0.4435 | 0.8265 | - | 5 | -23.738 | 62.5 | 0.58 | 0.231 |
| 5 | 0.6891 | - | - | - | - | 1.0300 | 4 | -26.546 | 64.2 | 2.27 | 0.099 |
| 4 | -1.0480 | - | - | - | 0.8928 | - | 4 | -27.187 | 65.5 | 3.56 | 0.052 |
| 12 | 0.6291 | - | 0.22840 | - | - | 1.1980 | 5 | -26.568 | 68.1 | 6.24 | 0.014 |
| 10 | 1.4020 | - | 0.02631 | 0.5349 | - | - | 5 | -26.670 | 68.3 | 6.44 | 0.012 |
| 21 | 0.9348 | - | 0.08490 | 0.5552 | - | 0.9572 | 6 | -24.645 | 68.9 | 7.03 | 0.009 |
| 2 | 1.1950 | - | 0.15840 | - | - | - | 4 | -28.966 | 69.0 | 7.11 | 0.009 |
| 20 | -1.0010 | - | 0.05731 | 0.4485 | 0.8803 | - | 6 | -24.870 | 69.4 | 7.48 | 0.007 |
| 11 | -1.0630 | - | 0.15080 | - | 0.8880 | - | 5 | -27.704 | 70.4 | 8.51 | 0.004 |
| 8 | -5.0710 | 1.401e-02 | - | - | 2.1110 | - | 5 | -28.728 | 72.5 | 10.56 | 0.002 |
| 7 | 1.6680 | -5.190e-03 | - | 0.5765 | - | - | 5 | -29.324 | 73.6 | 11.75 | 0.001 |
| 17 | 0.8493 | -6.239e-03 | 0.31060 | - | - | 1.4150 | 6 | -27.173 | 74.0 | 12.09 | 0.001 |
| 19 | 1.0800 | -2.230e-03 | - | 0.5623 | - | 0.8497 | 6 | -27.941 | 75.5 | 13.62 | 0.000 |
| 18 | -1.1230 | 1.806e-03 | - | 0.4160 | 0.8842 | - | 6 | -27.974 | 75.6 | 13.69 | 0.000 |
| 1 | 1.1280 | 1.339e-03 | - | - | - | - | 4 | -32.522 | 76.1 | 14.23 | 0.000 |
| 9 | 0.7403 | -1.520e-03 | - | - | - | 1.0590 | 5 | -30.041 | 76.2 | 14.33 | 0.000 |
| 16 | -5.2660 | 1.440e-02 | 0.11680 | - | 2.2190 | - | 6 | -29.480 | 78.6 | 16.70 | 0.000 |
| 15 | 1.8520 | -8.253e-03 | 0.12770 | 0.6084 | - | - | 6 | -30.041 | 79.7 | 17.82 | 0.000 |
| 6 | 1.1940 | 4.614e-05 | 0.15380 | - | - | - | 5 | -33.010 | 81.0 | 19.12 | 0.000 |
| Full | -1.0090 | -4.137e-05 | 0.12340 | 0.4449 | 0.7440 | 0.8240 | 8 | -27.290 | 86.6 | 24.68 | 0.000 |

**Supplementary Table 9** Results of phylogenetically controlled model selection for the relaxed estimate of pollen virome richness. Models are ranked by AICc value. Asterisks denote models of equal support that were averaged.

| **Model** | **(Intercept)** | **Number of sampled flowers** | **PC1** | **PC2** | **Pollinator diversity** | **Life history** | **df** | **logLik** | **AICc** | **delta** | **weight** |
| --- | --- | --- | --- | --- | --- | --- | --- | --- | --- | --- | --- |
| **Conditional average** | **2.8182** | **-** | **-** | **1.8941** | **2.5174** | **2.6143** | - | - | - | - | - |
| 13* | -1.0040 | - | - | 1.784 | 2.954 | - | 5 | -45.268 | 105.5 | 0.00 | 0.242 |
| 14* | 5.2150 | - | - | 2.345 | - | 3.515 | 5 | -45.697 | 106.4 | 0.86 | 0.158 |
| 5* | 4.8850 | - | - | - | - | 1.700 | 4 | -47.674 | 106.4 | 0.89 | 0.155 |
| 4* | 1.3450 | - | - | - | 1.717 | - | 4 | -47.834 | 106.7 | 1.21 | 0.132 |
| 3* | 6.4840 | - | - | 1.509 | - | - | 4 | -47.974 | 107.0 | 1.49 | 0.115 |
| 12 | 4.8890 | - | -0.0003462 | - | - | 1.675 | 5 | -47.121 | 109.2 | 3.71 | 0.038 |
| 2 | 5.6630 | - | -0.0873100 | - | - | - | 4 | -49.113 | 109.3 | 3.77 | 0.037 |
| 20 | -0.6546 | - | -0.0589100 | 1.729 | 2.811 | - | 6 | -44.976 | 109.6 | 4.05 | 0.032 |
| 11 | 1.5160 | - | 0.0148400 | - | 1.646 | - | 5 | -47.294 | 109.6 | 4.05 | 0.032 |
| 10 | 6.3680 | - | -0.2745000 | 1.425 | - | - | 5 | -47.448 | 109.9 | 4.36 | 0.027 |
| 21 | 5.7450 | - | -0.1037000 | 1.679 | - | 1.884 | 6 | -45.475 | 110.6 | 5.05 | 0.019 |
| 9 | 5.9350 | -0.03018 | - | - | - | 2.193 | 5 | -49.813 | 114.6 | 9.09 | 0.003 |
| 1 | 6.7770 | -0.02504 | - | - | - | - | 4 | -52.016 | 115.1 | 9.57 | 0.002 |
| 18 | 0.4880 | -0.01197 | - | 1.841 | 2.615 | - | 6 | -47.972 | 115.6 | 10.04 | 0.002 |
| 7 | 7.5850 | -0.02404 | - | 1.528 | - | - | 5 | -50.303 | 115.6 | 10.07 | 0.002 |
| 8 | 3.8400 | -0.02046 | - | - | 1.081 | - | 5 | -50.393 | 115.8 | 10.25 | 0.001 |
| 19 | 6.8290 | -0.02648 | - | 1.619 | - | 2.058 | 6 | -48.185 | 116.0 | 10.47 | 0.001 |
| 6 | 6.7670 | -0.02534 | -0.0654000 | - | - | - | 5 | -51.465 | 117.9 | 12.39 | 0.000 |
| 17 | 5.9470 | -0.03049 | 0.0426200 | - | - | 2.213 | 6 | -49.251 | 118.1 | 12.60 | 0.000 |
| 15 | 7.4700 | -0.02467 | -0.2584000 | 1.428 | - | - | 6 | -49.762 | 119.2 | 13.62 | 0.000 |
| 16 | 4.0310 | -0.02116 | 0.0057200 | - | 1.016 | - | 6 | -49.815 | 119.3 | 13.73 | 0.000 |
| Full | 1.1350 | -0.01520 | 0.1018000 | 1.833 | 2.156 | 1.633 | 8 | -45.649 | 123.3 | 17.76 | 0.000 |

**Supplementary Table 10** Pairs of pollen-associated viral taxa found in two different co-flowering plant species within the community determined to be the same virus or variants of one another since their RdRps are 100% identical or the percent identities between their RdRps are greater than the ICTV family-specific percent identity thresholds, respectively.

| **Virus family** | **Viruses** | **Plant species** | **Percent identity** | **E value^1^** |
| --- | --- | --- | --- | --- |

| *Amalgaviridae* | Mimulus guttatus amalgavirus 1  Mimulus nudatus amalgavirus 1 | *Mimulus guttatus*  *Mimulus nudatus* | 99.59 | 0.0 |
| --- | --- | --- | --- | --- |
| *Benyviridae* | Mimulus guttatus benyvirus 1  Mimulus nudatus benyvirus 1 | *Mimulus guttatus*  *Mimulus nudatus* | 95.92 | 0.0 |
| *Betaflexiviridae* | Apple stem grooving virus (novel variant 1)  Zigadenus venenosus betaflexivirus 1 | *Ranunculus californicus*  *Zigadenus venenosus* | 95.20 | 1E-102 |

| *Narnaviridae* | Ocimum basilicum RNA virus 2 (novel variant 1)  Ocimum basilicum RNA virus 2 (novel variant 2) | *Clarkia concinna*  *Lasthenia californica* | 100.00 | 0.0 |
| --- | --- | --- | --- | --- |
|  | Ocimum basilicum RNA virus 2 (novel variant 1)  Ocimum basilicum RNA virus 2 (novel variant 5) | *Lasthenia californica*  *Linanthus dichotomus* | 99.20 | 0.0 |
|  | Ocimum basilicum RNA virus 2 (novel variant 1)  Ocimum basilicum RNA virus 2 (novel variant 4) | *Lasthenia californica*  *Linanthus dichotomus* | 98.63 | 0.0 |
|  | Ocimum basilicum RNA virus 2 (novel variant 1)  Ocimum basilicum RNA virus 2 (novel variant 3) | *Eriophyllum lanatum*  *Lasthenia californica* | 73.81 | 2E-81 |
|  | Ocimum basilicum RNA virus 2 (novel variant 2)  Ocimum basilicum RNA virus 2 (novel variant 5) | *Clarkia concinna*  *Linanthus dichotomus* | 99.35 | 0.0 |
|  | Ocimum basilicum RNA virus 2 (novel variant 2)  Ocimum basilicum RNA virus 2 (novel variant 3) | *Clarkia concinna*  *Eriophyllum lanatum* | 77.88 | 9E-51 |
|  | Ocimum basilicum RNA virus 2 (novel variant 3)  Ocimum basilicum RNA virus 2 (novel variant 5) | *Eriophyllum lanatum*  *Linanthus dichotomus* | 72.31 | 2E-60 |
| No family | Mimulus guttatus no family 1  Mimulus nudatus no family 2 | *Mimulus guttatus*  *Mimulus nudatus* | 87.74 | 0.0 |
|  | Mimulus guttatus no family 1  Mimulus nudatus no family 3 | *Mimulus guttatus*  *Mimulus nudatus* | 86.41 | 0.0 |
|  | Mimulus guttatus no family 3  Mimulus nudatus no family 4 | *Mimulus guttatus*  *Mimulus nudatus* | 93.69 | 0.0 |
| *Partitiviridae* | Spinach cryptic virus 1 | *Agoseris heterophylla*  *Mimulus guttatus* | 100.00 | 0.0 |
|  | Agoseris heterophylla partitivirus 3  Eriophyllum lanatum partitivirus 4 | *Agoseris heterophylla*  *Eriophyllum lanatum* | 100.00 | 0.0 |
|  | Agoseris heterophylla partitivirus 6  Eriophyllum lanatum partitivirus 2 | *Agoseris heterophylla*  *Eriophyllum lanatum* | 100.00 | 0.0 |
|  | Calochortus luteus partitivirus 2  Eriophyllum lanatum partitivirus 3 | *Calochortus luteus*  *Eriophyllum lanatum* | 100.00 | 0.0 |
|  | Calochortus luteus partitivirus 2  Sidalcea diploscypha partitivirus 1 | *Calochortus luteus*  *Sidalcea diploscypha* | 100.00 | 0.0 |
|  | Eriophyllum lanatum partitivirus 3  Sidalcea diploscypha partitivirus 1 | *Eriophyllum lanatum*  *Sidalcea diploscypha* | 100.00 | 0.0 |
|  | Eschscholzia californica partitivirus 1  Lasthenia californica partitivirus 1 | *Eschscholzia californica*  *Lasthenia californica* | 100.00 | 0.0 |
|  | Eschscholzia californica partitivirus 1  Sisyrinchium bellum partitivirus 2 | *Eschscholzia californica*  *Sisyrinchium bellum* | 100.00 | 0.0 |
|  | Lasthenia californica partitivirus 1  Sisyrinchium bellum partitivirus 2 | *Lasthenia californica*  *Sisyrinchium bellum* | 99.81 | 0.0 |
| *Phenuiviridae* | Agoseris heterophylla phenuivirus 1  Lasthenia californica phenuivirus 1 | *Agoseris heterophylla*  *Lasthenia californica* | 87.44 | 0.0 |
|  | Agoseris heterophylla phenuivirus 1  Lasthenia californica phenuivirus 3 | *Agoseris heterophylla*  *Lasthenia californica* | 87.86 | 0.0 |
| *Secoviridae* | Calochortus luteus secovirus 3  Eriophyllum lanatum secovirus 2 | *Calochortus luteus*  *Eriophyllum lanatum* | 100.00 | 0.0 |
|  | Calochortus luteus secovirus 1  Clarkia gracilis secovirus 1 | *Calochortus luteus*  *Clarkia gracilis* | 99.76 | 0.0 |
|  | Calochortus luteus secovirus 1  Delphinium uliginosum secovirus 1 | *Calochortus luteus*  *Delphinium uliginosum* | 99.68 | 0.0 |
|  | Calochortus luteus secovirus 1  Eriophyllum lanatum secovirus 1 | *Calochortus luteus*  *Eriophyllum lanatum* | 99.66 | 0.0 |
|  | Clarkia gracilis secovirus 1  Delphinium uliginosum secovirus 1 | *Clarkia gracilis*  *Delphinium uliginosum* | 99.74 | 0.0 |
|  | Clarkia gracilis secovirus 1  Eriophyllum lanatum secovirus 1 | *Clarkia gracilis*  *Eriophyllum lanatum* | 99.91 | 0.0 |
|  | Delphinium uliginosum secovirus 1  Eriophyllum lanatum secovirus 1 | *Delphinium uliginosum*  *Eriophyllum lanatum* | 99.61 | 0.0 |
|  | Calochortus luteus secovirus 3  Lasthenia californica secovirus 1 | *Calochortus luteus*  *Lasthenia californica* | 99.41 | 0.0 |
|  | Eriophyllum lanatum secovirus 2  Lasthenia californica secovirus 1 | *Eriophyllum lanatum*  *Lasthenia californica* | 99.38 | 0.0 |

^1^E value: the number of better alignments that are expected to occur by chance; considered significant if < 0.001

**Literature cited**

Fetters AM, Cantalupo PG, Wei N, Sáenz Robles MT, Stanley A, Stephens JD, Pipas JM, Ashman T-L. 2022. The pollen virome of wild plants and its association with variation in floral traits and land use. Nat Commun 13: 523.

Fetters AM, Ashman T-L. 2023. The pollen virome: a review of pollen-associated viruses and consequences for plants and their interactions with pollinators. Am J Bot 110: e16144.

Ling K-S, Harrison HF, Simmons AM, Zhang SC, Jackson DM. 2011. Experimental host range and natural reservoir of sweet potato leaf curl virus in the United States. Crop Prot 30: 1055–1062.
